# Nanocomposite Hydrogel Films Based on Sequential Interpenetrating Polymeric Networks as Drug Delivery Platforms

**DOI:** 10.3390/polym15153176

**Published:** 2023-07-26

**Authors:** Gabriela Toader, Alice Ionela Podaru, Aurel Diacon, Edina Rusen, Alexandra Mocanu, Oana Brincoveanu, Mioara Alexandru, Florina Lucica Zorila, Mihaela Bacalum, Florin Albota, Ana Mihaela Gavrila, Bogdan Trica, Traian Rotariu, Mariana Ionita, Marcel Istrate

**Affiliations:** 1 Military Technical Academy “Ferdinand I”, 39-49 G. Cosbuc Blvd., 050141 Bucharest, Romania; nitagabriela.t@gmail.com (G.T.); podaru.alice04@gmail.com (A.I.P.); traian.rotariu@mta.ro (T.R.); 2 Faculty of Chemical Engineering and Biotechnologies, University Politehnica of Bucharest, 1-7 Gheorghe Polizu Street, 011061 Bucharest, Romania; alexandra.mocanu@upb.ro; 3 National Institute for Research and Development in Microtechnologies—IMT Bucharest, 126A Erou Iancu Nicolae Street, 077190 Bucharest, Romania; oana.brincoveanu@imt.ro; 4 Research Institute of the University of Bucharest, University of Bucharest, Soseaua Panduri, nr. 90, Sector 5, 050663 Bucharest, Romania; 5 Horia Hulubei National Institute of Physics and Nuclear Engineering, 30 Reactorului Street, 077125 Magurele, Romania; mioara.alexandru@nipne.ro (M.A.); florina.zorila@nipne.ro (F.L.Z.); bmihaela@nipne.ro (M.B.); florin.albota@nipne.ro (F.A.); 6 Department of Genetics, Faculty of Biology, University of Bucharest, 91-95 Splaiul Indepententei, 050095 Bucharest, Romania; 7 National Institute of Research and Development for Chemistry and Petrochemistry, 202 Splaiul Independentei, 060041 Bucharest, Romania; ana.gavrila@icechim.ro (A.M.G.); bogdan.trica@icechim.ro (B.T.); 8 Faculty of Medical Engineering, University Politehnica of Bucharest, Gheorghe Polizu 1-7, 011061 Bucharest, Romania; mariana.ionita@polimi.it; 9 Advanced Polymer Materials Group, University Politehnica of Bucharest, Gheorghe Polizu 1-7, 011061 Bucharest, Romania; 10 eBio-Hub Research Centre, University Politehnica of Bucharest-Campus, Iuliu Maniu 6, 061344 Bucharest, Romania; 11 S.C. Stimpex S.A., 46-48 Nicolae Teclu Street, 032368 Bucharest, Romania; office@stimpex.ro

**Keywords:** photopolymerization, hydrogel films, interpenetrating polymeric networks, drug delivery, nafcillin, covalent and ionic crosslinking

## Abstract

In this study, novel materials have been obtained via a dual covalent and ionic crosslinking strategies, leading to the formation of a fully interpenetrated polymeric network with remarkable mechanical performances as drug delivery platforms for dermal patches. The polymeric network was obtained by the free-radical photopolymerization of N-vinylpyrrolidone using tri(ethylene glycol) divinyl ether as crosslinker in the presence of sodium alginate (1%, weight%). The ionic crosslinking was achieved by the addition of Zn^2+^, ions which were coordinated by the alginate chains. Bentonite nanoclay was incorporated in hydrogel formulations to capitalize on its mechanical reinforcement and adsorptive capacity. TiO_2_ and ZnO nanoparticles were also included in two of the samples to evaluate their influence on the morphology, mechanical properties and/or the antimicrobial activity of the hydrogels. The double-crosslinked nanocomposite hydrogels presented a good tensile resistance (1.5 MPa at 70% strain) and compression resistance (12.5 MPa at a strain of 70%). Nafcillin was loaded into nanocomposite hydrogel films with a loading efficiency of up to 30%. The drug release characteristics were evaluated, and the profile was fitted by mathematical models that describe the physical processes taking place during the drug transfer from the polymer to a PBS (phosphate-buffered saline) solution. Depending on the design of the polymeric network and the nanofillers included, it was demonstrated that the nafcillin loaded into the nanocomposite hydrogel films ensured a high to moderate activity against *S. aureus* and *S. pyogenes* and no activity against *E. coli*. Furthermore, it was demonstrated that the presence of zinc ions in these polymeric matrices can be correlated with the inactivation of *E. coli*.

## 1. Introduction

Polymers are widely used in tablets and capsules as excipients because they provide sophisticated and advanced characteristics for controlled drug release [1]. For several decades, there has been a growth in the demand for polymers with superior properties, which led to the development of polymeric formulations with improved characteristics: tunable properties, biocompatibility, antimicrobial activity, good mechanical resistance, drug loading and release features, high versatility, etc. [2].

Hydrogels have been researched for various pharmacological, biological, and practical purposes, including drug delivery, contact lenses, tissue engineering, and superabsorbent agents. However, they are sometimes difficult to handle, may be difficult to load with medications and to sterilize, and often have limited mechanical strength.

Interpenetrating polymer networks (IPNs) [3] have demonstrated superior performances to traditional individual polymers, and as a result, the variety of applications for this class of materials has expanded rapidly [4]. These materials are an assortment of polymers combined into a network, where at least one is generated and/or crosslinked independently in the immediate vicinity of another polymer [1,5,6]. When polymer chains from the second system entangle with/or penetrate the network created by the first polymer, IPN is formed. Each polymeric network keeps its unique characteristics, allowing synergistic gains. [4].

IPNs are usually classified as either semi- or full IPNs based on whether one or both of their components are crosslinked [7]. Each component of a full IPN is present as a crosslinked network. The preparation method—simultaneous or sequential crosslinking of two polymers in a blend—is commonly used to describe the synthesis strategy of an IPN-structured material [8]. In sequential IPN, the polymerization of the second polymeric component occurs after the first polymer network components have been polymerized [1,6]. Among the many IPNs, sequential IPN is most suited for assembling an internally restrained control of the first network by the second [9]. Sequential IPNs provide a wide range of alternatives for synthesizing tunable materials because one network is synthesized after another [9].

According to several research studies published during the past few years, IPN-based delivery systems have emerged as a unique carrier in the controlled delivery of drugs [4]. Medication can be administered to patients by various routes [10], including oral, subcutaneous, vascular, mucosal, pulmonary, and transdermal delivery. Nevertheless, transdermal drug delivery remains to be preferred over traditional drug delivery systems due to benefits such as greater patient compliance and comfort due to a reduced dose frequency, a more efficient medication therapy for patients, and more consistent drug therapeutic levels [11].

The polymers selected for designing IPNs for the dermal administration of drugs must have adequate mechanical strengths, provide conditions for a sustained and controlled release of the medication, and demonstrate suitable biocompatibility. In contrast to the conventional materials used, IPN hydrogels are used to create even more sophisticated skin patches and bandages for wound care [12,13]. Dressings and patches are the first line of defense for injured tissues in hospitals, on the battlefield, or at home [14,15] to control bleeding and contamination from dirt and bacteria [16,17]. When applied to the wound, they come into direct contact with the soft tissues involved, where blood nutrients and exudate from the wound might encourage bacterial growth and biofilm formation [18,19]. Most bandages and patches are made of cotton, but some also involve using hydrogels or other materials that absorb water.

Biopolymers (e.g., alginate, chitosan), along with biocompatible synthetic polymers (e.g., polyvinyl alcohol (PVA), poly(*N*-vinylpyrrolidone) (PNVP), or polyethylene glycol (PEG)), have been extensively studied [20] and used to develop new materials for biomedical purposes. A valuable synthetic polymer that is water soluble and has numerous positive attributes, such as low toxicity, chemical stability, and strong biocompatibility, is poly(*N*-vinylpyrrolidone) [21]. Sodium alginate (Na-Alg) is one of the commonly used biodegradable polysaccharide, and alginate hydrogels are ideal candidates for various applications due to their mild synthesis, biocompatibility, and biodegradability [22]. IPN hydrogels based on sodium alginate offer a wide range of applications in extended and controlled drug release [10].

Wenbo Wang et al. reported the synthesis of pH-sensitive semi-IPN superabsorbent hydrogels based on sodium alginate-g-poly(sodium acrylate) and polyvinylpyrrolidone [23]. Yu-Mei Yue et al. developed a series of interpenetrating polymer network films of poly(acrylic acid) and poly(vinyl alcohol), describing the relationship between the composition and mechanical characteristics or swelling ratio of the IPN films [24]. M. Jose Moura et al. reported an in situ synthesis of chitosan hydrogels prepared via coupled ionic and covalent crosslinking [25]. Yiying Yue et al. described the synthesis of polyacrylamide: sodium alginate interpenetrating polymer-network-structured hydrogels, incorporating three types of inorganic nanoparticles (carbon nanotubes, nanoclays, nanosilicas) that exhibited excellent mechanical strengths and elasticities [22].

Whitin this framework, the challenge remains to design, for each situation, the optimal polymeric network with cohesive properties, the required adherence to types of surfaces, high and interlinked porosity, fluid retention capacity, selection, and the efficient loading of active ingredients with multi-purpose abilities, and at the same time maintain the biocompatibility, biodegradability, low toxicity, chemical stability, and mild conditions for tissular application. These considerations justify the conclusion that more investigation is required in this field to satisfy the many challenges imposed on the materials’ design and properties.

This study aimed to develop new nanocomposite hydrogel films that could serve as drug delivery platforms for dermal patches. Poly(N-vinylpyrrolidone) and sodium alginate were selected as suitable candidates for the hydrogel films to permit a double crosslinking strategy to be employed. Thus, the design of an IPN based on two independently crosslinked polymeric networks was selected as the system to grant the strength and resilience of the hydrogel. Thus, through the combination of covalent and ionic crosslinking of these IPNs, the synthesized hydrogel films revealed superior mechanical properties, possessing a homogenous and highly resistant structure. Bentonite nanoclay was incorporated in the hydrogel formulation to improve the mechanical performance and the absorption capacity of the hydrogel. Due to their potential for biomedical applications, TiO_2_ or ZnO nanoparticles were also included in two of the IPNs’ formulations.

To the best of our knowledge, this is the first study that addresses the synthesis and characterization of nanocomposite hydrogel films based on sequential interpenetrating polymeric networks, which merge the synergetic features brought by the combination of two organic polymer matrices (based on poly(*N*-vinylpyrrolidone) and sodium alginate), interconnected by both covalent and ionic crosslinking, with multifunctional nanosized fillers (bentonite, TiO_2_, ZnO) with a demonstrated capacity to act as a delivery platform for nafcillin.

This work showed that the herein-disclosed nanocomposite IPN hydrogel films possess suitable properties to serve as a platform for drug administration on cutaneous infections, and their manufacturing procedure implies a two-step, easy-to-follow procedure.

## 2. Materials and Methods

### 2.1. Materials

The materials employed in the synthesis process and used as received included: hydrophilic bentonite nano-clay (BT, Sigma-Aldrich, St. Louis, MO, USA), sodium alginate (ALG, from brown algae, Carl Roth, MW 300,000-350,000 g/mol), tri(ethylene glycol) divinyl ether (TEGDVE, Sigma Aldrich, St. Louis, MO, USA), 2-hydroxy-4′-(2-hydroxyethoxy)-2-methylpropiophenone (Ph-In, Sigma-Aldrich, St. Louis, MO, USA), zinc acetate dihydrate (Zn(CH_3_COO)_2_ 2H_2_O, Sigma-Aldrich, St. Louis, MO, USA), nafcillin sodium salt monohydrate (nafcillin, Sigma-Aldrich, St. Louis, MO, USA), and phosphate-buffered saline (PBS, Sigma-Aldrich, St. Louis, MO, USA). To remove the inhibitor, *N*-vinyl pyrrolidone (NVP, stabilized with *N,N*′-di-sec-butyl-1,4-phenylenediamine, Sigma Aldrich, St. Louis, MO, USA) was distilled at 92–95 °C (11 mmHg) and stored at cold until being employed for the synthesis of the hydrogels. TiO_2_ and ZnO nanoparticles were synthesized according to ref. [26] and ref. [27], respectively.

Materials used for the antimicrobial activity included: Mueller–Hinton broth (MHb) (Merck, Rahway, NJ, USA), Mueller–Hinton agar (Merck, Rahway, NJ, USA), brain–heart infusion broth (BHb) (Merck, Rahway, NJ, USA), brain–heart infusion agar (BHa) (Merck, Rahway, NJ, USA), 90 mm diameter Petri dishes, phosphate-buffered saline (PBS) (Merck, Rahway, NJ, USA). The bacteria strains were *Staphylococcus aureus* (ATCC 6538) as a model for Gram-positive bacteria; *Streptococcus pyogenes* ATCC 19615 as a potential skin contaminant; and *Escherichia coli* (ATCC 8739) as a model for Gram-negative bacteria [28]. *S. aureus* and *E. coli* were cultivated overnight in Mueller–Hinton broth at 37 °C with stirring (300 rpm). Then, the bacterial strains were harvested. Portions of the suspension were separated using a centrifuge and again suspended in PBS (Sigma-Aldrich, St. Louis, MO, USA). *S. pyogenes* was cultivated in brain–heart infusion broth overnight at 37 °C with stirring (300 rpm). The suspensions were adjusted to around 10^7^ CFU/mL [29]. The choice of the agar medium depends upon the type of microorganism being tested in the experiment. These recommendations have been defined by international organizations, such as the European Committee for Antimicrobial Susceptibility Testing [30] and the Clinical and Laboratory Standards Institute [31]. In general, the antimicrobial susceptibility of bacteria is tested on Mueller–Hinton agar [29].

For cytotoxicity evaluation, human foreskin cell line, BJ (ATCC, CRL-2522, Manassas, VA, USA), was cultured in DMEM (Dulbecco’s modified Eagle medium), supplemented with 10% fetal bovine serum (FBS) and penicillin–streptomycin (1%−100 units/mL), in a humidified atmosphere of 95% air/5% CO_2_ at 37 °C. All cell cultivation media and reagents were purchased from Biochrom AG (Berlin, Germany).

### 2.2. Methods

#### 2.2.1. Synthesis of Hydrogels

Step 1—Photopolymerization

To obtain the hydrogel films with a 3D network structure comprising covalent crosslinking sites, the first step involved free radical photopolymerization. The photocrosslinking process of the nanocomposite hydrogel films was possible using transparent glass molds (sealed with silicone rubber, 7 mm × 7 mm × 2 mm) and a UV lamp (low-pressure Hg UV lamp, λ_em_ = 254 nm). The nanofiller composition and the sample codes for the synthesized materials are detailed in Table 1. The solvent used for all the syntheses was water (deionized water). Sodium alginate was introduced as 2 wt.% aqueous solution, which was firstly prepared by dissolving sodium alginate in distilled water, under vigorous stirring, at 80 °C, for 4 h. Afterward, the required volume of sodium alginate solution was added to the main formulation in accordance with the values described in Table 1. For samples I and III, the monomer (monomer + ALG in the case of sample III), the crosslinker, and the photoinitiator were first dissolved in distilled water. For samples IV, IV.I, and IV.II, the nanofillers, BT, and TiO_2_/ZnO were first dispersed through ultrasonication, followed by the addition of the other reactants (monomer + ALG, crosslinker, and the photoinitiator). Then, the aqueous formulations were introduced into the glass molds (containers were 100% filled), and for 30 min, nitrogen gas was purged and evacuated via a two-syringe setup inserted across the silicone rubber seal into the reaction mixture. Subsequently, each side of the glass mold was exposed to UV light (through rotation every 5 min). The hydrogels’ UV-curing reaction was completed after approximately one hour.

Step 2—Ionic crosslinking

After the completion of the UV-curing, the hydrogel films were transferred in Petri dishes (Ø = 12 cm), each one containing 100 mL of an aqueous solution of zinc acetate (10 wt.%), to achieve supplementary ionic cross-linkages through the formation of ionic bonds between ALG units and Zn^2+^ ions retained within the hydrogel matrix. The nanocomposite hydrogel films were maintained in this solution until swelling equilibrium was reached.

#### 2.2.2. Antibiotic Loading/Release Tests

Nafcillin was introduced into the lyophilized nanocomposite hydrogels to examine the release characteristics of the active ingredient for each formulation. Our previous work [32] extensively details the procedures followed for monitoring the nafcillin loading and releasing processes from analogous hydrogels. Briefly, an aqueous nafcillin solution (5 × 10^−3^ M) was utilized for the loading of nanocomposite hydrogel samples weighing approximately 30 mg. Nafcillin release experiments were conducted in PBS (pH = 7.4, 37 °C). Recurrent samplings (1 mL) at predetermined time intervals were used to measure UV–Vis absorbance at 330 nm and correlate the values obtained with the corresponding nafcillin concentration. The release patterns of the nanocomposite hydrogels were plotted using data from the UV–Vis study and compared to evaluate and identify the mathematical model [32].

#### 2.2.3. Evaluation of the Anti-Microbiological Activity by Diffusion Method and Time–Kill Test and Biocompatibility Evaluation via Cytotoxicity Investigations

**Anti-microbiological properties of the hydrogel films**

For the microbiology assay, TiO_2_ and ZnO nanoparticles, the nanocomposite hydrogel films (before and after nafcillin loading) were separately investigated for antimicrobial activity.

In the first step, a preliminary evaluation of the antibacterial properties of the synthesized materials was performed through specific microbiological investigations: hydrogels were evaluated by *agar diffusion method*, and *time–kill test*. The nanoparticles (ZnO and TiO_2_) were tested independently to assess their minimal inhibitory concentration (MIC).

**Agar disk-diffusion testing** [33] is the official approach in many clinical microbiology laboratories for routine antimicrobial susceptibility testing. Agar plates are inoculated with a standardized inoculum (10^5^ CFU/mL) of the test microorganism. The agar surface is then covered with filter-paper discs (approximately 6 mm in diameter) containing the test chemical substances at the required concentration. The Petri dishes are incubated under appropriate conditions (36 °C for 24 h). Generally, an antimicrobial agent diffuses into the agar and inhibits the germination and growth of the test microorganism, and then the diameters of inhibition growth zones are evaluated. This method cannot differentiate bactericidal and bacteriostatic effects. Moreover, the agar disk-diffusion method is unsuitable for determining the minimum inhibitory concentration (MIC), as it is difficult to count the number of antimicrobial strains diffused into the agar medium. Chloramphenicol (30 µg/disk) was used as positive control. The antibacterial activity was assayed by measuring the diameter of inhibition zone (IZ) formed around the well.

**Time–Kill assay** [34] is the most accurate method for determining bactericidal effects. It is an appropriate method for obtaining information about the antimicrobial contaminant and microbial strain interaction. The Time–Kill test reveals a time-dependent or a concentration-dependent antimicrobial effect. The portions of 0.5 × 0.5 cm from each sample were placed in contact with 1 mL of inoculum from each type of microorganism (bacterial strain suspensions—10^7^ CFU/mL). The samples and inocula were mixed and vortexed to produce a homogeneous suspension. They were maintained in direct contact for 15 min, 30 min, and 1 h. At each set time interval, 0.1 mL of each sample was inoculated onto the agar surface and incubated at 37 °C for 24 h. The bacterial survivability was assessed following incubation.

**The minimal inhibitory concentration (MIC)** and minimal bactericidal concentration (MBC) methods were applied for the nanoparticles (ZnO and TiO_2_). The MIC value is defined as the lowest concentration of the antimicrobial agent that inhibits the visible growth of the microorganisms tested, usually expressed in mg/mL or mg/L. The MBC is defined as the lowest concentration of antibacterial agent needed to kill 99.9% of the final inoculum after incubation for 24 h under a standardized set of conditions [33]. The broth microdilution method established minimum inhibitory concentrations (MICs) [34,35]. Two-fold serial dilutions of each solution were performed in MHb. Negative and positive controls were associated [36]. The inhibitory effect of the powders was evaluated starting from a 4% concentration (the samples of substances were diluted 1:1 with MHb). A total of 10 µL of the microorganism suspensions (~10^4^ CFU) was added in each well, corresponding to the testing samples and controls. Because powders cause turbidity, the bacterial growth is difficult to discern, so at the end of the incubation period, 10 µL of resazurin 0.1% was added to each well. After 2 h of incubation with resazurin, the plates were read out.

According to the procedures described above, the nanocomposite hydrogel films (before loading nafcillin) and, separately, TiO_2_ and ZnO nanoparticles, were applied for the in vitro treatment of *Staphylococcus aureus* and *Escherichia coli* bacteria for microbiological investigations. The antimicrobial efficiency of the samples was compared with the control measurements, employing a disc with chloramphenicol.

In the second step, the five nafcillin-loaded nanocomposite hydrogel samples were applied for the in vitro evaluation against *Staphylococcus aureus*, *Streptococcus pyogenes,* and *Escherichia coli* bacteria. Their antimicrobial activity was evaluated by the *Agar disk-diffusion* method, which is suitable for samples like antibiotic-loaded hydrogels [32].

**Biocompatibility evaluation via cytotoxicity investigations**
**Sample preparation**

Two different evaluating conditions were used to evaluate their biocompatibility. Similar-sized shapes were cut, and the samples were briefly washed in PBS. For the first one, the washed samples were afterward directly used to assess their biocompatibility. For the second one, the washed samples were maintained in a cell growth medium for 5 days to observe if solvents (residual zinc acetate in this case) could be extracted from the material. For each condition, two different surfaces were used for MTT assay and one surface for microscopy images.

**Biological Evaluation**

Cell metabolic activity of BJ cells was assessed by MTT assay (Serva), as described previously [37]. The cells were seeded in 24-well plates at a density of 20,000 cells/well for MTT assay or on a 12 mm diameter cover glass for microscopy experiments. The samples were also placed in the same wells, while for control conditions, the cells were grown on glass slides in similar conditions. The samples were incubated for 24 h, after which the medium and the discs were removed, and 1 mg/mL MTT was added to each well diluted in a culture medium. The cells were incubated for 4 h, followed by the medium removed, and dimethyl sulfoxide (DMSO) was added to dissolve the crystals formed by the mitochondria. The solution absorbance was recorded at λ = 570 nm using the plate reader Mithras LB 940 (Berthold, Bad Wildbad, Germany). Cell viability was determined with the formula: (corrected absorbance of treated cells/corrected absorbance of control cells) ×100. The data were processed using the Prism 5.0 software. The morphological changes were investigated by fluorescence microscopy by staining the actin filaments with Phalloidin-FITC (Sigma-Aldrich, Saint Louis, MO, USA), and the nucleus with Hoechst 33342 (Invitrogen, Karlsruhe, Germany) for the samples where the viability was above 50%. The cells grown on a cover glass in the presence of the samples for 24 h were first washed with phosphate-buffered saline (PBS) three times. Afterward, the cells were fixed with 4% formaldehyde, rewashed three times with PBS, permeabilized with 0.1% Triton X-100 in PBS, and washed again with PBS three times. Finally, the two fluorescent dyes were added together on the cells and left in the dark, at room temperature, for 1.5 h. In the last step, the cells were washed with PBS three times and fixed with FluorSaveTM (Merck, Darmstadt, Germany). The fluorescence images were taken with a confocal microscope (Andor DSD2 Confocal Unit, Singapore) mounted on an Olympus BX-51 epifluorescence microscope, using a 40× objective. The images were recorded using a DAPI/Hoechst filter cube (excitation filter 390/40 nm, dichroic mirror 405 nm, and emission filter 452/45 nm) in the case of the nucleus and a GFP/FITC filter cube (excitation filter 466/40 nm, dichroic mirror 488 nm, and emission filter 525/54 nm) in the case of actin filaments. The images were further pseudo-colored and overplayed using the ImageJ software (version 1.53a, Madison, WI, USA).

### 2.3. Characterizations

FT-IR analysis was performed on a Spectrum Two FT-IR Spectrometer (Perkin Elmer, Waltham, MA, USA) with MIRacle™ Single Reflection A.T.R.—PIKE Technologies (Fitchburg, Wisconsin, USA), 32 scans, resolution 4 cm^−1^.

A high-resolution transmission electron microscope (HRTEM), model TECNAI F30 G2STWIN (Fei Company, Hillsboro OR, USA), was used at 300 kV acceleration voltage and with a resolution of 1 Å for TEM imaging of the nanofillers inside the hydrogels.

The morphological and structural characterization of the hydrogel samples were acquired via SEM-EDS with a Nova NanoSEM 630 scanning electron microscope (FEI Company, Hillsboro, OR, USA) at an acceleration voltage of 10 kV and an element energy dispersive spectroscopy (EDS) system (Smart Insight AMETEK) at an acceleration voltage of 10 kV. SEM images were captured on gold sputter-coated lyophilized hydrogels. Using SEM pictures, ImageJ software allowed for assessing porosity quantitatively [38]. The pore size distribution of samples IV, IV.I, and IV.II was extracted from SEM images by measuring around 250 individual pores.

Inductively coupled plasma mass spectrometry measurements were performed using an ICP-MS 7700s, Agilent Technologies, Santa Clara, CA, USA, having the following instrumental parameters: plasma gas Ar (6.0 or 99.9999 analytical purity) 15 L/min Ar; carrier gas: 0.7 L/min Ar; makeup gas: 0.5 L/min Ar; nebulizer pump: 0.1 RPS; collision gas (octopole reaction cell): 4 mL/min He. Mass spectrometer tuning was performed according to the manufacturer’s specifications, with a multielement standard solution—called tuning solution (5 elements in digestion matrix (nitric acid 5% and hydrochloric acid 0.5%): Li, Co, Y, Ce, and Tl, 5 ppb each)—in both “no gas” and “He” modes (in the collision cell—an octopole), calibrating the mass axis in 3 points for small, medium, and high masses, and optimizing the ion optics for the best signal-to-noise ratio. The data acquisition parameters of the mass spectrometer used: integration time 0.9 s per channel or 2.7 s per *m*/*z* (3 channels out of 20 available were monitored). Monitored masses (natural isotopic abundance, %): ^27^Al (100%), ^45^Sc—internal standard abbreviated ISTD (100%), ^47^Ti (7.44%), ^48^Ti (73.22%), ^64^Zn (48.63%), ^66^Zn (27.90%), ^66^Zn(18.75%), ^89^Y—ISTD (100%), ^209^Bi—ISTD (100%). Calibration standards were acquired to represent the calibration curves for each isotope (6 concentrations per element: 0.0001%, 0.001%, 0.01%, 0.1%, 1%, and 10% of the spike concentration, in order, from the most diluted calibration standard to the most concentrated one. Samples were measured afterward, running 2 wash blanks between two consecutive samples to prevent “memory effects” (carry-over). All the labware used for this study, made of perfluoralkoxy alkane (PFA), were thoroughly cleaned before use, following washing cycles with nitric acid vapors, ultrapure water leaching, a washing cycle with ultrapure water vapors, ultrapure water leaching, rinsing, and final drying at 50 °C. A matrix solution of 5% + 0.5% nitric and hydrochloric acids in water (all ultrapure), was prepared by using ultrapure nitric and hydrochloric acid, produced by Merck, Germany. Mono-element 1000 mg/L Reference Material solutions produced by Merck, Germany, were used for the preparation of the spike solution (see Table S1 from the Supplementary Materials file). This solution includes the following elements: Al, Ti, and Zn. Sc, Y, and Bi were used separately to prepare an internal standard solution.

The mechanical properties of the hydrogel films obtained through photopolymerization (followed by ionic crosslinking) were investigated on a Discovery 850 DMA TA Instruments analyzer in three different setups: shear sandwich setup (for the evaluation of the frequency-dependent shear modulus), tensile test setup (using a tensile clamp, designed for uniaxial deformation investigations) and compression setup (using the compression clamps kit). For shear sandwich analysis, two equal-size square-shaped slices of the same material (12 mm × 12 mm × 2 mm) were sheared between two fixed plates and a moving plate to measure shear modulus. In shear sandwich mode, a compressive pre-strain of 1% was applied to the hydrogels to obtain good adherence between the samples and the clamps. Frequency was logarithmically increased from 10^−1^ to 10^1^ Hz. On the same device (Discovery 850 DMA TA Instruments, New Castle, DE, USA), tensile clamps were further installed. Tensile tests were performed on rectangular samples (40 mm × 5 mm × 2 mm), at 5 mm/min. Similar tensile tests were performed on five specimens from each type of hydrogel film, and the mean values were reported. For convenience, only one curve from each set, whose parameters are closest to the mean values, is shown in the charts in the ‘Results and Discussion’ section. The compression clamps (φ = 40 mm) were installed on the same DMA instrument mentioned above to analyze the compressive strength of the synthesized hydrogels. The specimens, swollen at equilibrium, that were used for the compression tests, had a disc-shaped form, with diameters ~11.9 mm to 13.7 mm and thickness varying between 6.5 mm and 6.9 mm. Before compression tests, the hydrogel disks were maintained in zinc acetate solution to ensure the formation of additional ionic crosslinking, and they were regularly weighed until reaching their swelling equilibrium. Then, the hydrogel disks were submitted to a compression test in their equilibrium swollen state. The hydrogel discs were analyzed at a constant compression rate of 2 mm/min until fragmentation of the hydrogel occurred. Compression measurements were performed on five specimens from each sample. To improve readability, the ‘Results and Discussion’ plots only depict one curve from each set, whose parameters are closest to the mean values. The swelling degree (SD) of the nanocomposite hydrogels was calculated according to refs. [39,40].

A GBC Cintra 101 UV–Vis equipment was used to conduct the UV–Vis analysis (to evaluate the loading efficiency and the nafcillin release profile), with a resolution of 5 nm, in the wavelength range of 300 to 800 nm (reading the maximum absorption value at 330 nm).

**Statistical analysis**

The datasets were evaluated using Origin software (version 2018). The data were analyzed for a paired comparison of individual data means by one-way ANOVA analysis. A *p*-value < 0.05 was considered statistically significant. All the experiments were conducted three times and data were represented in mean ± SD.

## 3. Results and Discussion

### 3.1. Method Principle

**Figure 1:**
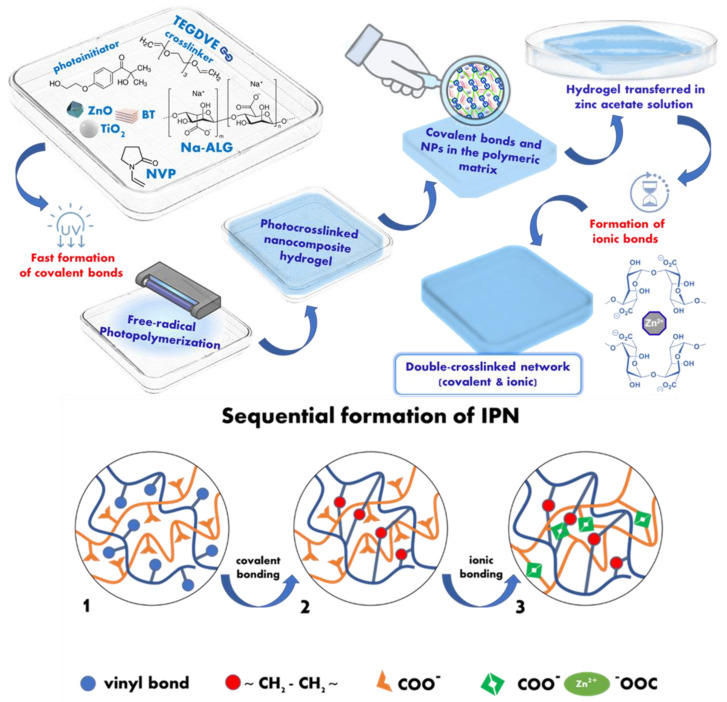
Schematic illustration of the sequential formation of IPN hydrogel films.

The hydrogels films developed in this study involve two organic polymeric matrices (based on poly(*N*-vinylpyrrolidone) and sodium alginate), interconnected by both covalent and ionic crosslinking (Figure 1), and nanosized fillers (bentonite, TiO_2_, and ZnO). The composition of these hydrogel films is based on sequential interpenetrating polymeric networks: the first step includes covalently PNVP crosslinked sequences interconnected by a TEGDVE crosslinker synthesized via free-radical photopolymerization in the presence of sodium alginate, while the second step consisted of the immersion of these films in zinc acetate solution for 24 h to allow sodium alginate chains to form additional crosslinking sites in the presence of Zn^2+^ ions. Hence, through these supplementary crosslinks, the mechanical resistance of the hydrogel films can be improved, while the presence of zinc may ensure a better antimicrobial efficacy.

### 3.2. FT-IR Analysis

**Figure 2:**
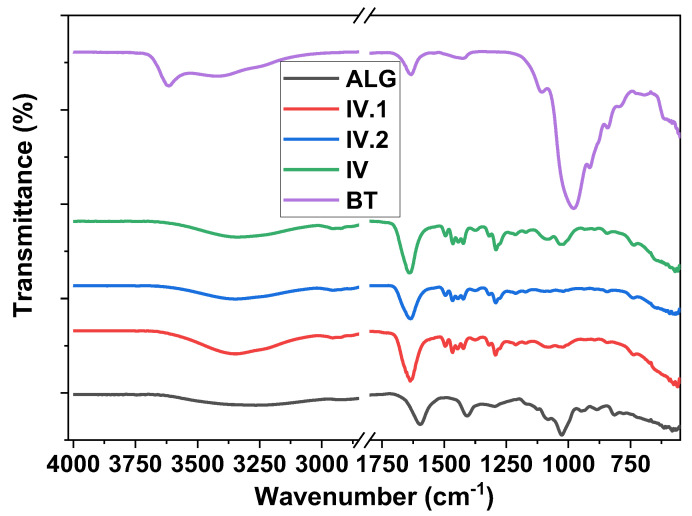
FT-IR spectra containing comparative plots for BT, ALG, and IV, IV.I, and IV.II nanocomposite hydrogels.

FT-IR analysis was performed to evaluate the interactions between the components of the composite hydrogel films. Figure 2 illustrates the comparative FT-IR analysis plots of materials obtained. The infrared spectrum of neat ALG displayed a broad peak around 3300 cm^−1^, which can be assigned to the O–H stretching from the carboxylic groups of alginates. Compared to the FT-IR spectra of the hydrogels, the O–H stretching peak exhibited slight variations in intensity and shifting toward 3340–3350 cm^−1^, probably due to the participation of these O–H groups in the interactions established inside the IPN between the components [41]. The bands at 1603 and 1409 cm^−1^ in the neat ALG, assigned to asymmetric and symmetric stretching peaks of carboxylate salt groups [42], respectively, are no longer visible on the spectra of the hydrogel, where other peaks specific for the >C=O stretching vibration of the amide from *N*-vinyl-2-pyrrolidone structure are visible around 1640 cm^−1^ [41]. New peaks that can be related to PNVP appeared in all the FT-IR spectra of hydrogels: C–H stretching and bending vibrations are marked by weak wide signals in the region 2800–3000 cm^−1^ and weak sharper signals in the region 1420–1490 cm^−1^; and the peak assigned to C–N group appeared at 1293 cm^−1^. The intensity of the characteristic peaks of bentonite [43] around 3415 cm^−1^ (Si–O–H stretching), the sharp peak situated at 980 cm^−1^ (Si–O–Si stretching), 918 cm^−1^ (Al–O–H bending) [44], and 790 cm^−1^ (Si–O–Al bending) were considerably reduced, or some peaks even disappeared in the hydrogel spectra probably due to the interactions established between the nanoclay and the polymeric network, and also due to the low concentration of nanofiller inside the polymeric matrix. The peaks specific for C–N and C–O stretching, at 1020 cm^−1^ and 1082 cm^−1^, respectively, are less visible, having a lower intensity in the spectra of the hydrogels containing TiO_2_ and ZnO nanoparticles.

### 3.3. Transmission Electron Microscopy—Energy Dispersive X-ray Analysis (TEM-EDX) Imaging

The synthesized materials were next subjected to TEM-EDX analysis to highlight the size and morphology of the nanofillers inside the polymeric matrix (Figures S1 and S2—from the Supplementary Materials file). The dark, multilamellar-shaped spots in the micrographs presented in Figures S1A,C and S2A,C can be associated with the hydrophilic clay phase [45]. The thinner, dark, isolated lines suggest the presence of some individual clay sheets from the intercalated or exfoliated morphology of bentonite. In contrast, the thicker ones may be assigned to a few agglomerates formed by clay nanoparticles or clay sheets. We can presume that the hydrophilic nature of the NVP monomer and its compatibility with BT allowed some of the PNVP chains to grow between the layers [46] of this hydrophilic clay. Figures S1A–C and S2A–D display TiO_2_ and ZnO nanoparticles, respectively. According to these TEM images, TiO_2_ and ZnO nanoparticles seem to have comparable dimension ranges, the main difference between them consisting of their shape. When comparing the TEM images of samples IV.II and IV.I, it can be observed that ZnO nanoparticles seem to be irregularly shaped and smaller than TiO_2_ nanoparticles, which possess a rounded shape and slightly larger dimensions. The EDX plots illustrated in Figures S1E and S2E—from the Supplementary Materials file—display the specific peaks for the representative elements (Ti, Zn, Si) of each type of nanoparticle present in the hydrogels.

### 3.4. Scanning Electron Microscopy (SEM) with Energy Dispersive X-ray Analysis (EDX)

#### 3.4.1. SEM Imaging

**Figure 3:**
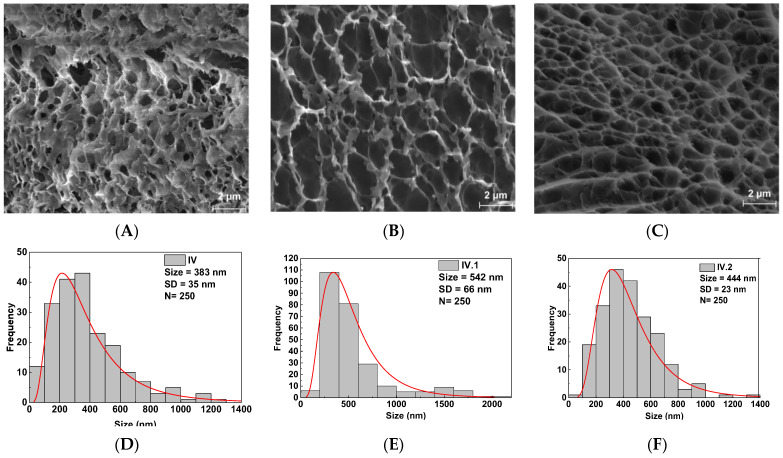
SEM images and pore size distribution of the synthesized hydrogel films: (**A**,**D**) sample IV; (**B**,**E**) sample IV.I; (**C**,**F**) sample IV.II.

SEM investigations offered evidence of the morphology of the lyophilized nanocomposite hydrogel films. The SEM images and the pore size distribution of samples IV, IV.I, and IV.II are illustrated in Figure 3. From these images, it can be concluded that the morphology and the pore size varied depending on the type of nanofiller employed for the synthesis, probably due to the distinct interactions between the components of the IPN structure and the nanoparticles. The size distribution of the pores was extracted from SEM images by measuring around 250 individual pores for each sample. The histograms were best fitted with the Gauss function to evaluate the pore size distribution, exhibiting a unimodal distribution of the IV, IV.I, or IV.II samples. The pore sizes of IV were found to range from 62 nm to 1298 nm. The highest frequency was found within the 118–400 nm diameter pore size range with a mean diameter of 383 ± 35 nm. The area that covers pores in the image is 22.7%. The IV.I pore size was found to range from 143 nm to 2086 nm. The highest frequency was found within the 191–590 nm diameter pore size range with a mean diameter of 542 ± 66 nm. The area covering pores in the image is 56.8%. The IV.I pore sizes, determined based on SEM analysis, were found to range from 93 nm to 1332 nm. The highest frequency was found within the 250–480 nm diameter pore size [47] range with a mean diameter of 444 ± 23 nm. The area that covers the pores in the image is 43.7%.

The pore size variation follows the same trends as in our previous work [32]. However, in this case, the pore sizes are considerably smaller due to the double crosslinking strategy adopted in this study. Thus, the alginate units permit the additional ionic crosslinking of the 3D IPN network of the hydrogel films.

#### 3.4.2. EDX Mapping

A uniform distribution of the nanosized components inside the hydrogels was achieved, as observed in EDX mapping in Figures S4–S6 from the Supplementary Materials file. However, in some areas, the zinc involved in the ionic bonds with alginate units are visible as denser regions on EDX mapping, following the pathway of the ionic crosslinking sites. In contrast, even if the presence of TiO_2_ nanoparticles inside the synthesized hydrogels was confirmed by TEM-EDAX analysis, Ti was not visible on EDX mapping, most likely because of the high radical scavenger capacity of TiO_2_ [48], which may have contributed to a greater level of polymeric wrapping surrounding these nanoparticles.

Further, the ICP-MS technique was utilized to obtain complementary information regarding the concentration of the nanofillers inside the IPN hydrogel films.

### 3.5. Inductively Coupled Plasma Mass Spectrometry (ICP-MS)

**Figure 4:**
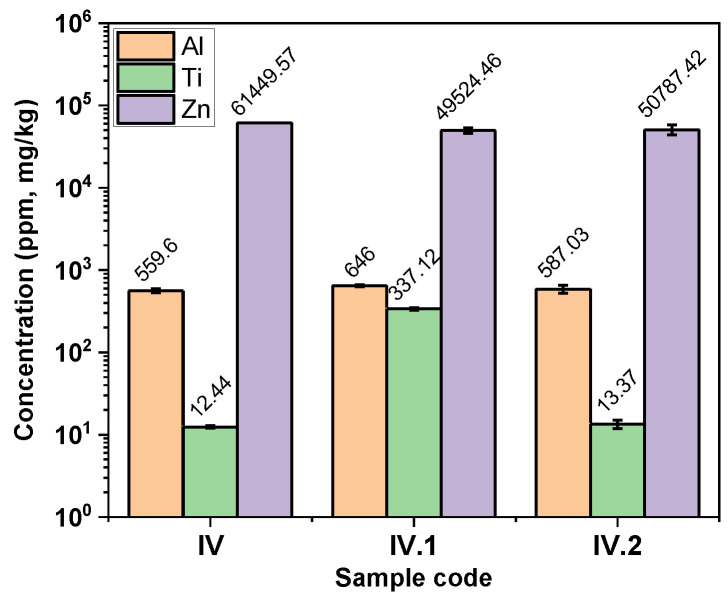
Comparative plot of the results obtained from ICP-MS.

The ICP-MS technique was used to track the abundance of the appropriate representative elements for each type of nanofiller contained in the composite hydrogel films, as follows: Al—for bentonite, Ti for TiO_2_, and Zn for both ZnO nanofiller and the Zn uptake from the zinc acetate solution. Figure 4 illustrates a comparative plot based on the results obtained for each sample, detailed in Table S2—from the Supplementary Materials file. ICP-MS offered evidence on the concentration of bentonite by monitoring the Al content found in the nanocomposite hydrogel films, and it can be observed that Al levels are relatively similar in all the analyzed samples (IV, IV.I, and IV.II). The presence of Ti was observed in the IV.I sample, which confirmed the presence of the polymer-coated TiO_2_ nanoparticles, which was assumed after the EDX analysis. Zinc presence was registered in all hydrogel samples because, in the sequential synthesis step, the covalently photocrosslinked hydrogels were further immersed in a zinc acetate solution to achieve the additional ionic bonding of the IPNs. According to the values obtained from ICP-MS analysis, it can be presumed that Zn concentration varied in accordance with the uptake capacity of the hydrogels (Figure S6—from the Supplementary Materials file). These values can be further correlated with the pore sizes of the samples (Figure 3) and connected with the swelling data obtained (Figure S6). Sample IV had the highest amount of Zn, probably due to its higher swelling capacity ensured by the multiple small pores. In contrast, Zn concentration was lower in sample IV.I, which had the larger pores and the lowest swelling capacity. Even if sample IV.II is the only one that contains ZnO, Zn concentration was lower in sample IV.II than in sample IV, most probably due to the higher swelling capacity of sample IV. Nevertheless, the Zn concentration values found via ICP-MS in all the nanocomposite hydrogel films had the same order of magnitude for all three samples tested. The differences that appeared could be related to their swelling degree at equilibrium (Figure S6).

### 3.6. Mechanical Analyses

**Figure 5:**
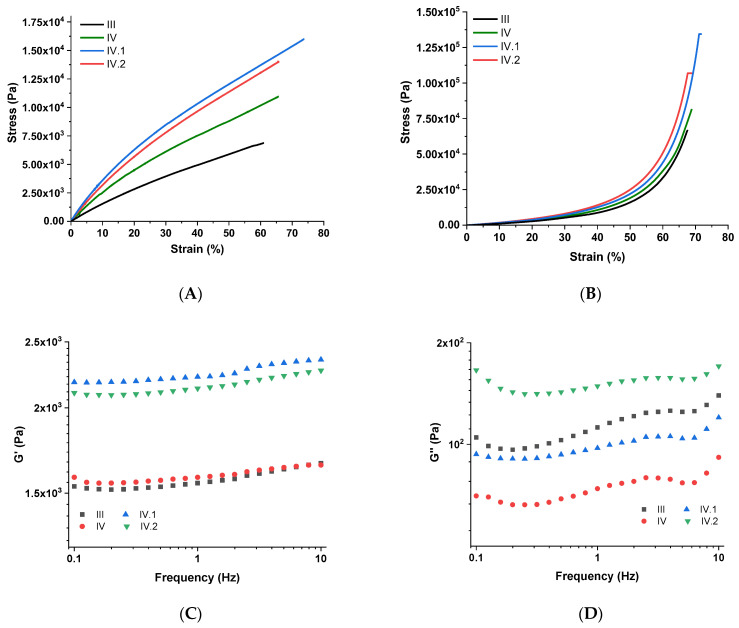
Mechanical properties of the nanocomposite hydrogel films: (**A**) tensile test; (**B**) compression test; (**C**,**D**) shear test.

The mechanical properties of the nanocomposite hydrogel films were evaluated through tensile, compression, and shear tests, and the results are illustrated in Figure 5.

Tensile test results showed that the unique design of these IPN hydrogel films provides them with strength and flexibility. A comparative tensile stress–strain plot is illustrated in Figure 5A. In contrast to the results obtained in our previous work [32], in this study, the hydrogel films comprise an additional ionic crosslinked polymeric matrix (ALG crosslinked via Zn^2+^ ions), whose presence ensured a superior mechanical resistance for analogous samples. When comparing samples III and IV (Figure 5A), it can be confirmed that the bentonite nanoclay positively influenced the mechanical resistance to the uniaxial loading of sample IV. As can be observed from Figure 5A, the hydrogels containing TiO_2_ (sample IV.I) and ZnO (sample IV.II) revealed superior mechanical performances, apparently owing to the good dispersion of these nanoparticles, which induced a supplementary reinforcing effect to these hydrogels.

Uniaxial compression has been employed to establish the compression strength of the fully swelled hydrogels, and Figure 5B illustrates the results obtained. Under compressive loading, the mechanical resistance of the samples maintained approximately the same trend as for tensile strength measurements. In compression mode, the differences between the samples are minor; the influence of each nanofiller on the mechanical resistance is no longer as visible, probably because they also demonstrated some slight differences in their swelling capacity (sample III vs. samples IV, IV.I, and IV.II). Regardless, compared to the analogous hydrogels developed in our previous study [32], in this case, the introduction of ALG led a strengthening effect on the hydrogels, which was also confirmed by a compression test. This time, better mechanical properties were achieved due to the supplementary crosslinking sites inside the 3D IPN network, comprising two polymeric matrices interconnected by two distinct types of bonding (covalent and ionic).

To evaluate the viscoelastic properties of the nanocomposite hydrogels, a “shear sandwich” setup was employed for assessing the frequency-dependent viscoelastic moduli. The results of this analysis are plotted in Figure 5C,D. Because the timeframe of the input stress decreased with increasing frequency, the polymeric chains inside the IPN had less time to relax, making their response progressively more elastic. As a result, the storage modulus (G′) increased with frequency. G′ > G″ also denotes a higher degree of elastic behavior than viscoelastic response. The slow increase in G’ values (Figure 5C) indicates that when these hydrogel films are subjected to shear stress, they will achieve a relatively fast relaxation of their polymeric network. In the case of samples IV.I and IV.II, the strengthening effects induced by TiO_2_ and ZnO suggest that the nanofiller was well dispersed throughout the IPN hydrogels. Due to the presence of various types of dynamic units in their networks, each of the synthesized hydrogels may also be differentiated by the amount of energy dissipated when shear stress is applied, denoted by G″ (Figure 5D). The values of G″ are usually directly influenced by the physical entanglements of the polymeric chains and the crosslinking density [49,50]. The efficiency with which these hydrogels release energy is dependent on the rate at which the contained dynamic bonds or entanglements are broken, recombined, or migrated, which, in response, impacts the resulting G″ values. A drop in G″ indicates that the nanocomposites were less effective at dissipating the energy because there were restricted conditions for the polymeric network to relax, whereas an increase in G″ usually indicates that the samples had more time to flow, depending on the motion of the dynamic units inside the material.

### 3.7. Drug Loading/Release

The nafcillin loading efficiency at pH 7.4 is presented in Figure S7. Given that the synthesized hydrogel in this study comprises PNVP as a primary/major component, in terms of drug loading capacity, the results obtained can be compared to other examples in the literature. The largest advantage of this polymer is the capacity to load both hydrophilic and hydrophobic drugs [51]. The simplest and the most commonly employed drug-release method is by diffusion [52]. For example, the drug release from the crosslinked pectin/PVP hydrogel membrane was studied by using salicylic acid (SA) as a model drug [53]. The maximum swelling was obtained at an alkaline pH (pH 7.4) of pectin/PVP hydrogels as compared to acidic pH (pH 1.4). This is attributed to the highly hydrophilic nature of PVP and the complete dissociation of carboxymethyl groups (COOCH_3_) present in pectin at a higher pH [53]. In another recent study [54], hydrogel based on PVP grafted with crotonic acid was used for the loading of ketoprofen. In this case, the maximum swelling degrees were achieved between pH 6 and 7 due to a complete dissociation of acidic groups of the crotonic acid groups at this pH value [54]. In the case of a hydrogel containing chitosan and PNVP [55], it was observed that the loading of amoxicillin takes place faster in an acidic medium than neutral or basic.

**Figure 6:**
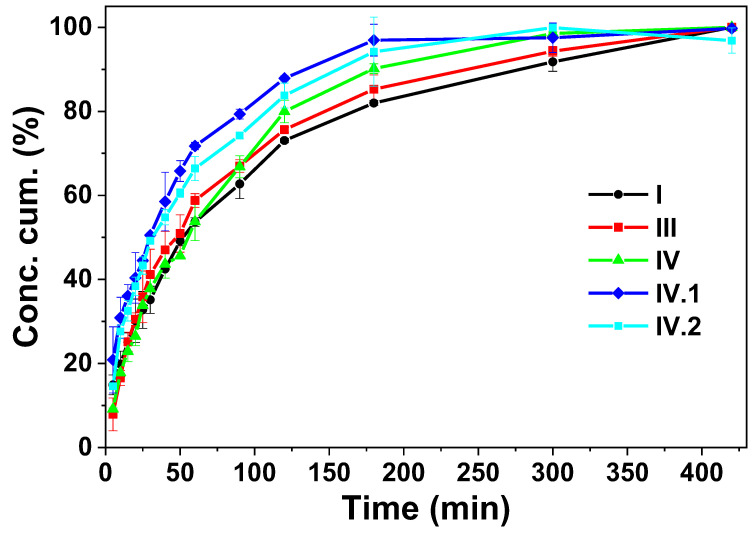
Cumulative concentration vs. time for nafcillin release in PBS solution.

**Scheme 1:**
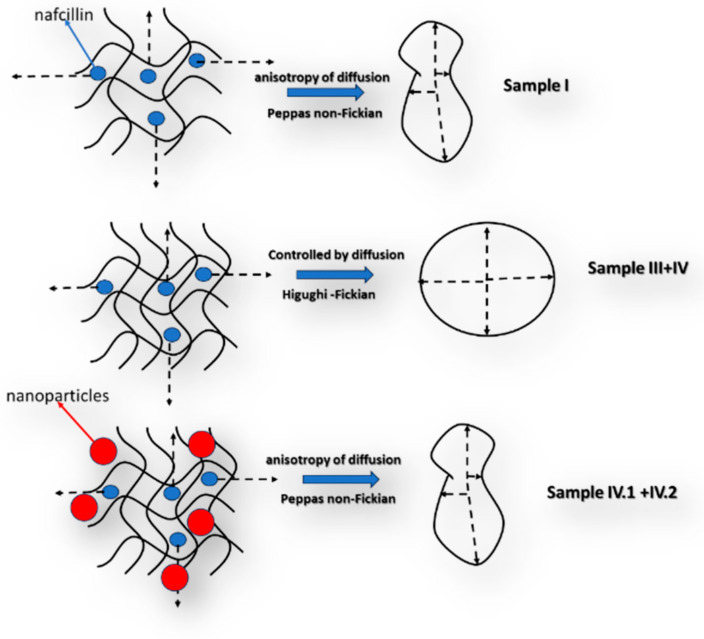
Schematic representation of the diffusion process during the drug release.

The drug release profile (Figure 6 and Scheme 1) is best depicted by mathematical models that describe the physical processes taking place during the drug transfer from the polymer to the solution. The most utilized models are: zero order, first order, Higuchi, and Peppas [56].

The comparison between the characteristics of the samples reveals that both the swelling degree and pore size diameter vary between them, which can determine relatively different release behaviors to be observed. In this context, in the case of sample I, based on the experimental data, the best fitting was obtained for the Peppas model with a value for the n coefficient of less than 0.5 (Table 2 and Figure S8), which can be explained by a non-Fickian release process. This means that the diffusive flow is not proportional to the concentration gradient. This behavior can be explained by the high swelling degree of this sample in comparison to the other samples, respectively, by a relatively poor solubility of the active substance in the release environment. In the case of samples III and IV, the mathematical model that describes the processes is Higuchi, the process being mainly controlled by the Fickian diffusion steps. Thus, the release behavior of the hydrogels was altered due to the addition of alginate, which was crosslinked with zinc acetate. Also, in the hydrogel, it is possible for an ionic exchange between the nafcillin, which is a sodium salt, and the zinc ions to take place. This process can control the mechanisms of diffusion that becomes Fickian. In the case of samples IV.I and IV.II, the best fitting was obtained using the Peppas model, again, a behavior explainable by the relative low swelling degree in the aqueous media, which can be attributed to the supersaturation effect of the nanoparticles on the polymeric matrix [57,58,59,60].

### 3.8. Anti-Microbiological Properties of the Hydrogel Films and Their Components

For the evaluation of the anti-microbiological properties of the nanocomposite hydrogel films, considering the nature and potential uses of these materials (solid samples, with potential cutaneous applications as “platforms” for antimicrobial agents in skin patches or wound dressings) instead of utilizing filter-paper discs in our experiments, we placed the disc-shaped samples directly on the microbial strains.

**Agar disk diffusion—no drug loaded in the nanocomposite hydrogel films**

For a preliminary screening of the antimicrobial activity of the samples, no drug was loaded inside the nanocomposite hydrogel films. Samples consisting of hydrogel discs (without nafcillin) were applied directly on the inoculated agar with *S. aureus* and *E. coli*. An inhibition area should be visible around the sample on the agar surface if the samples possess any antibacterial activity. Nevertheless, in this agar disk-diffusion screening, the hydrogel samples tested (before antibiotic loading) did not reveal an antimicrobial activity, possibly because the nanoparticles did not diffuse into the agar medium or maybe their concentration was too low to exercise an efficient inhibition on the growth of microorganisms.

**Time–Kill assay—no drug loaded in the nanocomposite hydrogel films**

For the time–kill assay, after the proposed contact times (15 min, 30 min, and 1 h) between strains and samples IV, IV.I, and IV.II, the bacterial growth was evaluated. In the case of the time–kill assay, it was observed that the antimicrobial activity was too small to be quantified after the abovementioned microbial strain–sample contact times. The sample containing ZnO (IV.II) revealed a weak activity after one hour against both microorganisms tested. Even so, since the decrease in the growth of microorganisms was only one log_10_, we cannot affirm that the samples have a significant antimicrobial activity (details in Table S3 from the Supplementary Materials file).

**MIC and MBC for TiO_2_ and ZnO nanoparticles**

MIC and MBC methods were utilized to evaluate the antimicrobial activity of TiO_2_ and ZnO nanoparticles to establish if they might influence the growth of the tested microorganisms. Thus, this preliminary screening of the antimicrobial activity of the nanosized components aimed to evaluate their MIC and MBC (Figure S9—from the Supplementary Materials file). These investigations revealed that ZnO NPs powder exhibited a low antimicrobial activity against bacterial strains used in the test: MIC 0.25% against *S. aureus* and 0.125% against *E. coli*. TiO_2_ NPs powder revealed no antimicrobial activity against Gram-negative (*E. coli*) or Gram-positive bacteria (*S. aureus*). MBC was not detected for any powder in the tested concentrations.

**Agar disk-diffusion test on nafcillin-loaded samples**

According to the literature, nafcillin is efficient against Gram-positive cocci and staphylococci that produce penicillinase [62]. This antibiotic primarily affects Gram-positive bacteria by having an inhibitory effect on them and functioning through the inhibition of cell wall biosynthesis as the main method of action. It inhibits the transpeptidase and carboxypeptidase activities by binding to penicillin-binding proteins, conferred by these proteins, and prevents the formation of the crosslinks [63]. In the case of Gram-negative bacteria, it does not exert this action.

Antibiotics perform better at treating bacterial infections when combined with active nanoparticles [64,65]. It is highly challenging for bacteria to develop resistance to nanoparticles because they operate against bacterial cells in several ways at once. The antibacterial activity of nanoparticles varies according to their type: nanoparticles like TiO_2_ and ZnO affect bacterial cells mostly by binding the bacterial cells and generating ROS [64,66]. The bacterial response to the activity exerted by nanoparticles is also influenced by a series of factors like their number, shape, charge, size, and their mode of action. The bacterial natural antioxidant defense system that deals with stress is also crucial [64].

The zinc ions usually induce an antimicrobial response due to the interference in metabolic processes and disturbance in enzymatic systems [67,68]. Through the adsorption of ZnO nanoparticles to the bio-membrane-via-charge interaction, or ROS generation under UV and visible light irradiation, ZnO also influences the antimicrobial response [69,70,71].

In agar disk-diffusion method, the patch samples revealed different antibacterial activities against the three selected microorganisms (Table 3).

**Figure 7:**
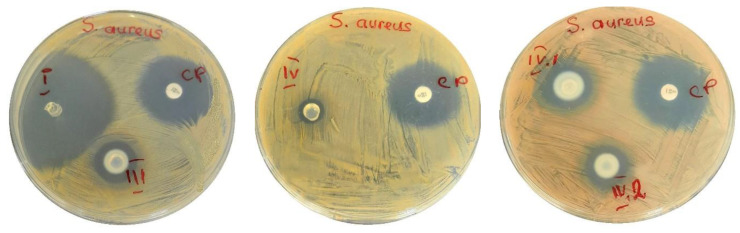
Disk-diffusion method showing the susceptibility of *S. aureus*—samples: I, III, IV, IV.I, and IV.II (CP = disc with chloramphenicol).

Thus, against *S. aureus* (Figure 7): from a strong activity in the case of sample I, to a medium activity obtained for samples III, IV.I, and IV.II, to a very thin ring of inhibition that suggests a very low susceptibility obtained for sample IV, compared to the reference antibiotic used as positive control (chloramphenicol). These results can be determined by the action of the active components: antibiotic (nafcillin) and nanoparticles (TiO_2_ and ZnO) and, also, due to zinc acetate.

Even if samples I and IV showed similar nafcillin loading efficiencies, the main difference between them is related to the number and type of crosslinking sites inside these IPNs (sample I has only covalent crosslinks while sample IV has both covalent and ionic crosslinking sites) which considerably influenced the efficacy for *S. aureus.*

The antibacterial effect noticed in the case of samples IV.I and IV.II is higher than the one of sample IV, probably due to the presence of TiO_2_ and ZnO nanoparticles and possibly also due to their faster release capacity, which can ensure providing a “shock-dose” of nafcillin to the tested strains. Compared to sample IV, sample III has a higher loading efficiency and a higher release rate; therefore, the antimicrobial activity against *S. aureus* of sample III was comparable with the ones of samples IV.I and VI.2.

**Figure 8:**
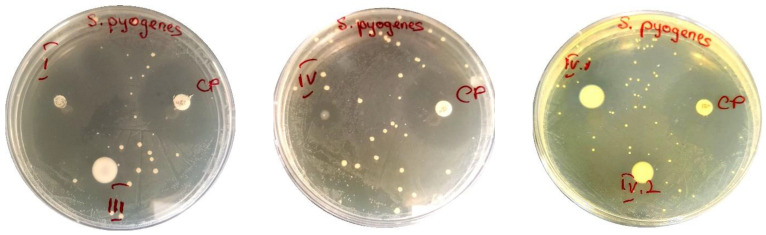
Disk-diffusion method showing the susceptibility of *S. pyogenes*—samples: I, III, IV, IV.I and IV.II (CP = disc with chloramphenicol).

Different antibacterial actions were noticed against *S. pyogenes* (Figure 8), from strong activity (sample I) to medium (samples IV and IV.I) or very weak (samples III and IV.II). This indicates that the interactions established by the same type of antibiotic-loaded nanocomposite hydrogel with a distinct type of bacterial strain can also strongly influence the antibacterial activity, leading to different results.

**Figure 9:**
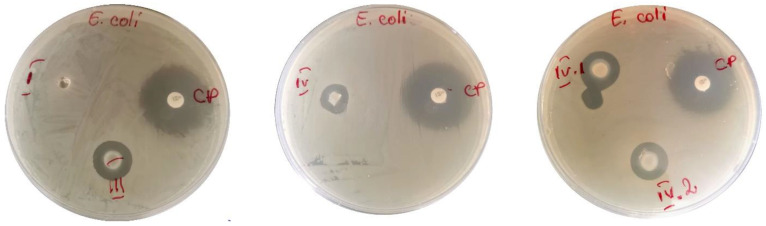
Disk-diffusion method showing the susceptibility of *E. coli*—samples: I, III, IV, IV.I, and IV.II (CP = disc with chloramphenicol).

Against *E. coli* (Figure 9)*,* the samples showed medium activities (samples III, IV, IV.I, and IV.II), while sample I did not reveal any inhibitory effect. It is well known that nafcillin is an antibiotic usually prescribed for staphylococcal infections, so we expected to find very weak or no activity against *E. coli* for sample I. But what is more interesting here is that the antibacterial effect of zinc (from zinc acetate utilized for ionic crosslinking) can be highlighted through this experiment. As can be observed from Figure 9, only samples III, IV, IV.I, and IV.II exhibited a bactericidal effect for the tested strains.

We can notice that around patches III, IV, IV.I, and IV.II, a medium-sized area of inhibition is obtained, compared to that obtained around the reference antibiotic (CP). Chloramphenicol (the reference antibiotic drug) showed an antibacterial activity against the Gram-negative and Gram-positive bacteria tested. The intensity of the inhibitory effect can be influenced by the amount of zinc released into the culture medium or by the interaction established by the microbial strains with the nanoparticles that migrated toward the microorganism.

### 3.9. Cytotoxicity Investigations

Since herein described nanocomposite hydrogels could be developed as a platform for cutaneous drug delivery, cytotoxicity investigations were performed to evaluate if the synthesized materials have any cytotoxic effect on a human skin cell line.

**Figure 10:**
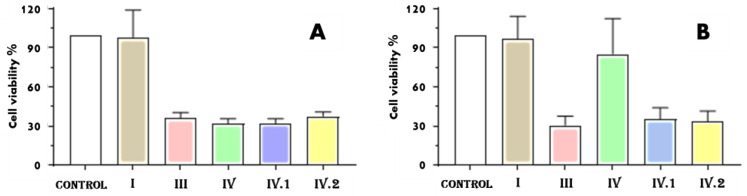
Cell viability in the case of BJ cells grown for 24 h in the presence of the nafcillin-loaded nanocomposite hydrogels investigated: (**A**) washed in PBS and (**B**) washed in PBS and kept for 5 days in DMEM.

The cell viability for the five nafcillin-loaded samples, treated in two different conditions (A washed in PBS, and B washed in PBS and kept for 5 days in DMEM), is presented in Figure 10.

Cell viability for sample I was not changed by either type of testing condition compared to control cells (Figure 10A,B), indicating that sample I is biocompatible independent of the method used to wash it. Considering the fact that the sample has nafcillin in its composition (a semisynthetic penicillin that usually shows low toxicity and side effects, except rare hepatic complications, but a high efficiency against methicillin-resistant staphylococcus aureus by innate immune system enhancement [72,73]), since cell viability was comparable to one of the control sample, it can be affirmed that nafcillin loading in the hydrogel samples proved to be noncytotoxic. In the first testing mode (when the samples were only washed in PBS, Figure 10A), all the other four nafcillin-loaded samples (additionally crosslinked through immersion in zinc acetate) had a cell viability around 30%. However, when these samples (III, IV, IV.I, and IV.II) were kept in the medium for 5 days (Figure 10B), the viability of the cells grown in the presence of sample IV exhibited a viability of 85.34%, indicating that, in this case, the sample is biocompatible. Based on the literature, biomaterials are considered biocompatible if the percentage of viable cells is ≥70% compared to the untreated cells [74].

Samples IV, IV.I, and IV.II have bentonite in common, but this nanoclay is well known for its biocompatibility, and it is used to prepare various medical devices or delivery systems, so it should not be considered when studying the influence of the components on the cytotoxicity of the samples [75].

The presence of the TiO_2_ or ZnO nanoparticles could have caused a lower cell viability. Yet, for samples IV.I and IV.II, the cytotoxicity did not exceed the one of sample III, so we cannot affirm that TiO_2_ or ZnO nanoparticles significantly influenced the cell viability.

The utilization of zinc acetate could explain the higher cytotoxicity observed after cells were grown with the samples for 24 h. Data reported in a recent study showed that the ZnO nanoparticles obtained starting from zinc acetate have a higher toxicity against blood cells compared with the ones obtained through green synthesis [76].

The cytotoxicity could also be related to the amount of zinc trapped in the hydrogel network during swelling in zinc acetate. The fact that the cell viability increased in the second trial for sample IV suggests that a significant amount of zinc was washed because it was not strongly bonded to the polymeric network, which did not occur for samples IV.I or IV.II, where only a slight increase in cell viability was observed. However, by applying different washing steps, the toxicity can be reduced.

**Figure 11:**
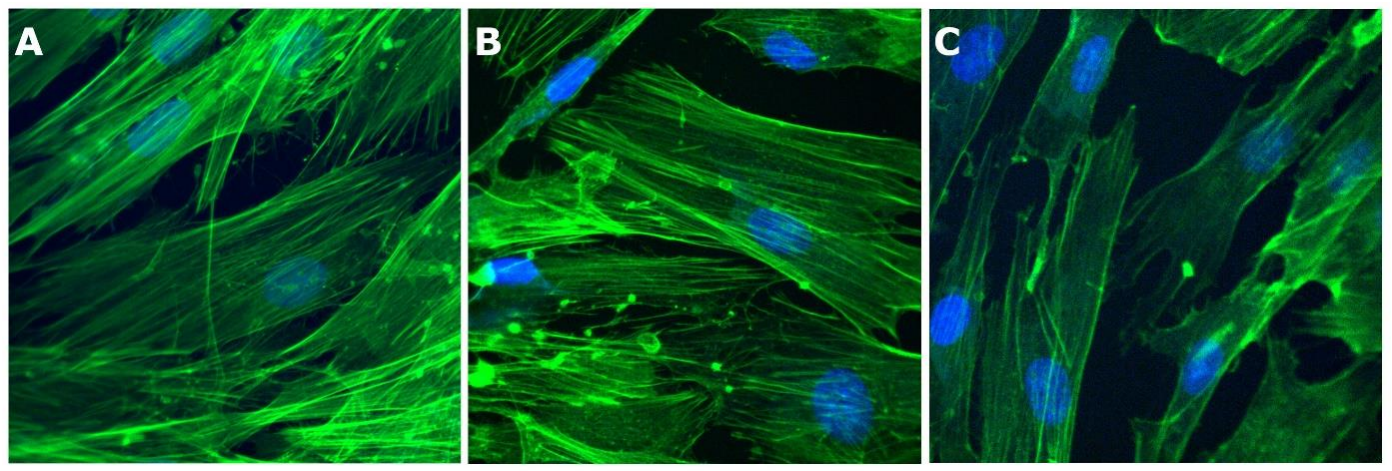
Fluorescence microscopy (40× objective) images of BJ cells grown on cover glass in the presence of the samples investigated: (**A**) control cells, (**B**) cells grown in the presence of sample I, and (**C**) cells grown in the presence of sample IV.

We also performed microscopy experiments to evaluate BJ cell morphology for the conditions where the viability was above 50% (Figure 11).

From Figure 10, it can be observed that BJ cell morphology for control cells is similar to data reported previously [37]. BJ control cells exhibit an elongated morphology, with actin filaments almost parallel, stretching from one end to the other end of the cell (Figure 11A). Regarding the nucleus, one can see that it has a specific oval morphology. A slightly reduced number was observed for the cells grown in the presence of samples I and IV. However, similar morphological characteristics are found. The data confirm that the tested samples did not induce any changes in the structure of BJ cells.

Based on the results obtained for the five samples, we can conclude that some traces of zinc acetate can cause toxicity to healthy cells following the preparation steps [76]. The purification procedure after synthesis can be tuned to reduce the cytotoxicity by subjecting the hydrogel films to successive washing protocols before nafcillin loading, to ensure a higher cell viability while maintaining antimicrobial activity.

## 4. Conclusions

The main physicochemical and morphological characteristics of the synthesized double-crosslinked IPN-based hydrogels were investigated through specific characterization techniques to determine the difference between the samples and the influence of the nanofillers. SEM images revealed the aspect and the size distribution of the pores of the lyophilized samples, while EDX mapping confirmed the uniform distribution of the nanoparticles along the hydrogel films. The ionic crosslinking pathways were also visible on some EDAX mapping images because some denser zinc areas were highlighted. ICP-MS helped quantify the zinc content from the samples after zinc acetate swelling reached equilibrium and confirmed the presence of the other types of nanoparticles inside the polymeric network.

The investigation of the mechanical properties of the synthesized 3D IPN hydrogels revealed that the additional crosslinking of alginate units with Zn^2+^ improved their mechanical resistance. The homogenous distribution of the nanofillers in the hydrogel also had a positive effect on the mechanical properties of the hydrogel films.

Nafcillin loading and release profiles were investigated via UV–VIS. The release profile was compared to mathematical models to better understand if the drug transport follows a Fickian or non-Fickian diffusion process. Agar disk-diffusion tests revealed different antibacterial activities against *S. aureus*, from strong activity for sample I, to medium activity obtained for samples III, IV.I, and IV.II, to a very low susceptibility obtained for sample IV. Different antibacterial activities were detected against *S. pyogenes* from strong activity (samples I) to medium (samples IV and IV.I) or very weak (samples III and IV.II). This suggests that interactions between a particular bacterial strain and a specific type of antibiotic-loaded nanocomposite hydrogel might also significantly affect the antibacterial activity and produce diverse outcomes. *E. coli* samples III, IV, IV.I, and IV.II exhibited a medium antibacterial activity, probably due to the zinc acetate and nanoparticles, which can lead to an “in situ” effect against this bacterial strain.

Based on cytotoxicity findings, to ensure a higher cell viability while maintaining antibacterial activity, the purification step following synthesis can be modified to reduce cytotoxicity by subjecting the hydrogel films to consecutive washing protocols before nafcillin loading.

This study demonstrated that the herein-reported nanocomposite hydrogel films possess favorable attributes to serve as a platform for drug delivery for cutaneous infections, while their synthesis process implies straightforward and accessible methods.

## Figures and Tables

**Table 1:** Sample codification and composition.

Sample Code	BT, wt.% *	TiO_2_, wt.% *	ZnO, wt.% *	ALG wt.% *	NVP wt.% *	TEGDVE wt.% *	Ph-In wt.% *
I	-	-	-	-	20	1.45	0.15
III	-	-	-	1	20	1.45	0.15
IV	0.2	-	-	1	20	1.45	0.15
IV.1	0.2	0.1	-	1	20	1.45	0.15
IV.2	0.2	-	0.1	1	20	1.45	0.15

* From the total weight of the reaction mixture, including water as solvent; BT—hydrophilic bentonite nano-clay; ALG—sodium alginate; NVP—N-vinyl pyrrolidone; TEGDVE—tri(ethylene glycol) divinyl ether; Ph-In—(photoinitiator)—hydroxy-4′-(2-hydroxyethoxy)-2-methylpropiophenone.

**Table 2:** Mathematical models fitting the nafcillin release profile.

Mathematical Models [32,61]	I	III	IV	IV.I	IV.II
**Zero order** $Q\%=k_{0}t$					
*k*_0_, min^−1^	0.3863	0.4175	0.4565	0.4212	0.4197
R^2^	0.9127	0.8547	0.9209	0.8583	0.859
**First order** $Q\%=1-e^{k_{1}t}$					
*k*_1_, min^−1^	1.0087	1.0207	1.0105	1.0074	1.0081
R^2^	0.7660	0.6809	0.6922	0.7051	0.6443
**Higuchi** $Q\%=k_{H}t^{0.5}$					
*k_H_*, min^−0.5^	6.3294	7.7739	7.4525	7.0395	7.0162
R^2^	0.9897	0.9791	0.9911	0.9683	0.9696
**Korsmeyer–Peppas** log⁡(Q%)=log⁡(k)+nlog(t)					
*n*	0.5011	0.7439	0.6244	0.4448	0.4958
R^2^	0.9947	0.9551	0.9812	0.9847	0.9646

Where: *k*_0_ is the zero-order release constant in units of concentration %/time, *k*_1_ is the first-order release constant, *k_H_* is the Higuchi dissolution constant, *k* is the kinetic constant characteristic of the drug-polymer system, and *n* is the release coefficient that indicates the type of diffusion mechanism.

**Table 3:** Zones of inhibition (mm) of bacteria growth by the nafcillin-loaded samples and reference antibiotic (chloramphenicol).

Name of Sample	Zones of Inhibition (mm) of Bacteria Growth		
	*E. coli*	*S. aureus*	*S. pyogenes*
Chloramphenicol (Reference antibiotic)	26.7 ± 0.12	23.7 ± 0.12	26.3 ± 0.12
I	Nil	36.00	38.00
III	15.00	12.00	Nil
IV	16.00	9.00	8.50
IV.I	20.00	14.00	16.00
IV.II	15.00	12.00	6.00

Values for chloramphenicol are presented as mean ± standard deviation of triplicate for each microorganism, Nil = zero.

## Data Availability

Not applicable.

## Supplementary Materials

The following supporting information can be downloaded at: https://www.mdpi.com/article/10.3390/polym15153176/s1, Figure S1—TEM images of bentonite layers (A,C) and TiO_2_ nanoparticles (A,B,D) and EDX spectra (E) of the hydrogel film IV.1; Figure S2—TEM images of bentonite layers (A,C) and ZnO nanoparticles (A–D) and EDX spectra (E) of the hydrogel film IV.2; Figure S3—SEM-EDX mapping of sample IV; Figure S4—SEM-EDX mapping of sample IV.1; Figure S5—SEM-EDX mapping of sample IV.2; Figure S6—Swelling degree at equilibrium; Figure S7—Nafcillin loading efficiency at pH 7.4; Figure S8—Nafcillin release - data fitting for samples I, III, IV, IV.1 and IV.2; Figure S9—MIC determination observed from broth microdilution assay using MH broth and resazurin; Table S1—ICP-MS accuracy and spike concentrations; Table S2—Concentrations of the representative elements in the nanocomposite hydrogels; Table S3—Number of CFU when no drug was loaded in the nanocomposite hydrogel films; Table S4—Minimal inhibitory concentration (MIC) and Minimal bactericidal concentration (MBC) values.
