# Supplementary material for: Nanocomposite Hydrogel Films Based on Sequential Interpenetrating Polymeric Networks as Drug Delivery Platforms
Source: Polymers (Basel). 2023 Jul 26;15(15):3176. doi: 10.3390/polym15153176 (PMC10420953; doi:10.3390/polym15153176)
Supplement: Supplementary file 1 [file polymers-15-03176-s001.zip › polymers-2509070-supplementary.pdf]

Supplementary materials

Article

Nanocomposite Hydrogel Films Based on Sequential Interpenetrating Polymeric Networks as Drug Delivery Platforms

Gabriela Toader [1,†], Alice Ionela Podaru [1,2,†], Aurel Diacon [1,2,*], Edina Rusen [2,*], Alexandra Mocanu [2,3], Oana Brincoveanu [3,4,], Mioara Alexandru [5], Florina Lucica Zorila [5,6], Mihaela Bacalum [5], Florin Albota [5], Ana Mihaela Gavrila [7], Bogdan Trica [7], Traian Rotariu [1], Mariana Ionita [8,9,10] and Marcel Istrate [11]

[1] Military Technical Academy "Ferdinand I", 39-49 G. Cosbuc Blvd., 050141 Bucharest, Romania; nitagabriela.t@gmail.com (G.T.); podaru.alice04@gmail.com (A.I.P.); traian.rotariu@mta.ro (T.R.)
[2] Faculty of Chemical Engineering and Biotechnologies, University Politehnica of Bucharest, 1–7 Gheorghe Polizu Street, 011061 Bucharest, Romania; alexandra.mocanu@upb.ro
[3] National Institute for Research and Development in Microtechnologies—IMT Bucharest, 126A Erou Iancu Nicolae Street, 077190 Bucharest, Romania; oana.brincoveanu@imt.ro
[4] Research Institute of the University of Bucharest, University of Bucharest, Soseaua Panduri, nr. 90, Sector 5,
050663 Bucharest, Romania
[5] Horia Hulubei National Institute of Physics and Nuclear Engineering, 30 Reactorului Street, 077125 Magurele, Romania; mioara.alexandru@nipne.ro (M.A.); florina.zorila@nipne.ro (F.L.Z.); bmihaela@nipne.ro (M.B.); florin.albota@nipne.ro (F.A.)
[6] Department of Genetics, Faculty of Biology, University of Bucharest, 91-95 Splaiul Indepententei,
050095 Bucharest, Romania
[7] National Institute of Research and Development for Chemistry and Petrochemistry, 202 Splaiul Independentei, 060041 Bucharest, Romania; ana.gavrila@icechim.ro (A.M.G.); bogdan.trica@icechim.ro (B.T.)
[8] Faculty of Medical Engineering, University Politehnica of Bucharest, Gheorghe Polizu 1-7, 011061 Bucharest, Romania; mariana.ionita@polimi.it
[9] Advanced Polymer Materials Group, University Politehnica of Bucharest, Gheorghe Polizu 1-7, 011061 Bucharest, Romania
[10] eBio-Hub Research Centre, University Politehnica of Bucharest-Campus, Iuliu Maniu 6, 061344 Bucharest, Romania
[11] S.C. Stimpex S.A., 46-48 Nicolae Teclu Street, 032368 Bucharest, Romania; office@stimpex.ro
* Correspondence: aurel_diacon@yahoo.com (A.D.); edina_rusen@yahoo.com (E.R.)
† Co-first authors, equally contributed to this study.

Table of Contents

Table S1- ICP-MS accuracy and spike concentrations 2
Figure S1 - TEM images of bentonite layers (A, C) and TiO_2 nanoparticles (A, B, D) and EDX spectra (E) of the hydrogel film IV.1 3
Figure S2 - TEM images of bentonite layers (A,C) and ZnO nanoparticles (A, B, C, D) and EDX spectra (E) of the hydrogel film IV.2 4
Figure S3 – SEM-EDX mapping of sample IV 5
Figure S4 – SEM-EDX mapping of sample IV.1 6
Figure S5 – SEM-EDX mapping of sample IV.2 7
Table S2 – Concentrations of the representative elements in the nanocomposite hydrogels 8
Figure S6 – Swelling degree at equilibrium 8
Figure S7 – Nafcillin loading efficiency at pH 7.4 .... 8s
Figure S8 – Nafcillin release - data fitting for samples I, III, IV, IV.1 and IV.2 11

Table S3 - Number of CFU when no drug was loaded in the nanocomposite hydrogel films 14
Table S4 - Minimal inhibitory concentration (MIC) and Minimal bactericidal concentration (MBC) values 14
Figure S9 - MIC determination observed from broth microdilution assay using MH broth and resazurin 14

Characterization

ICP – MS

The method's accuracy is between 93% and 114%, and the acceptance limit is (70-150%) with precision under 14% and the acceptance limit 20%. The internal standard used for normalization was 209Bi since it is not present in the measured samples and has a variation of 8.9%.

Table S1- ICP-MS accuracy and spike concentrations

	Al	Ti	Zn
Accuracy	93.27%	97.89%	114.09%
Concentration (ppm, mg/kg)	280.26	314.44	308.54

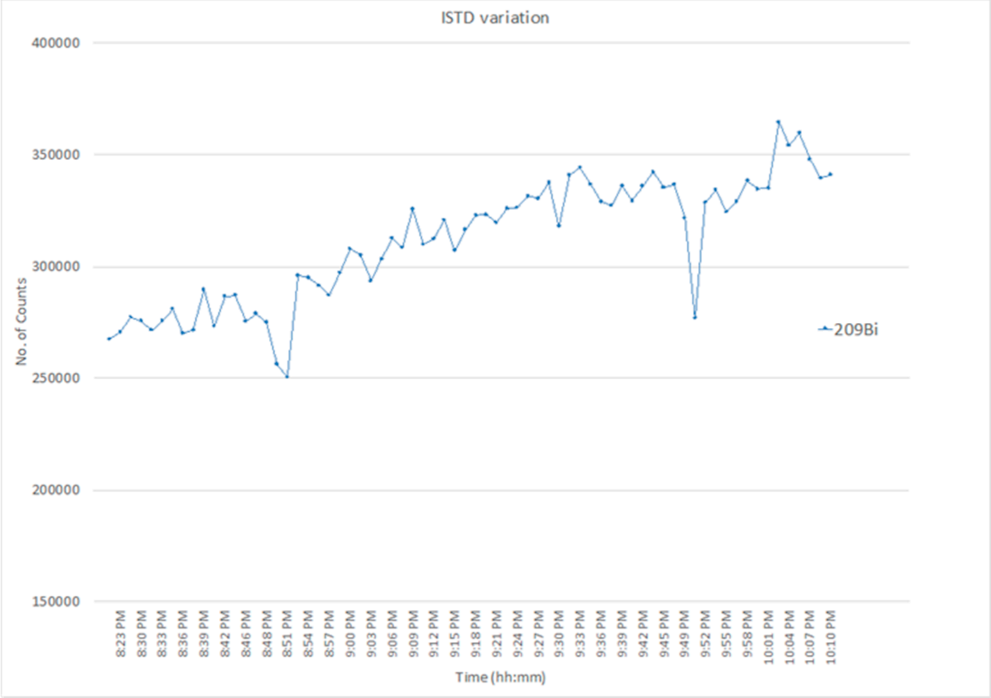

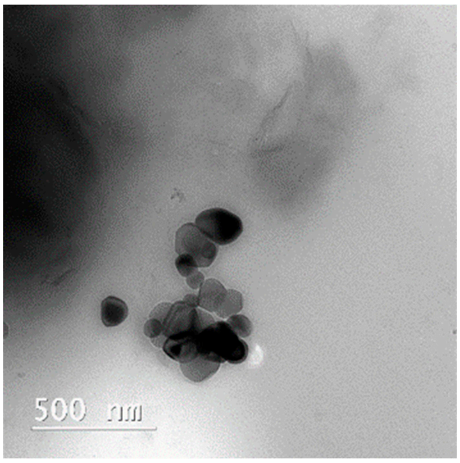

A

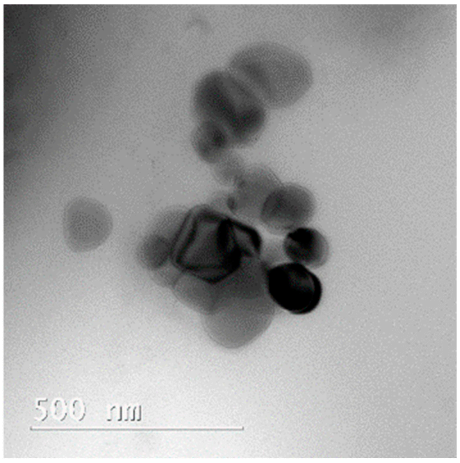

B

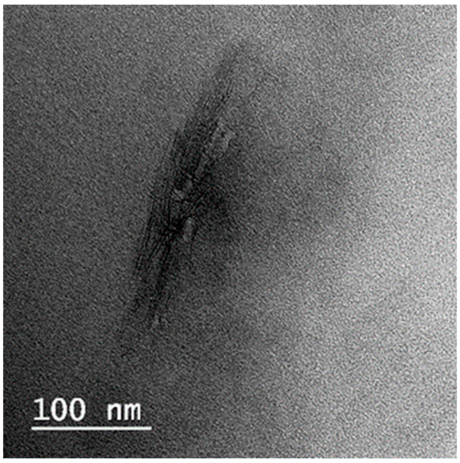

C

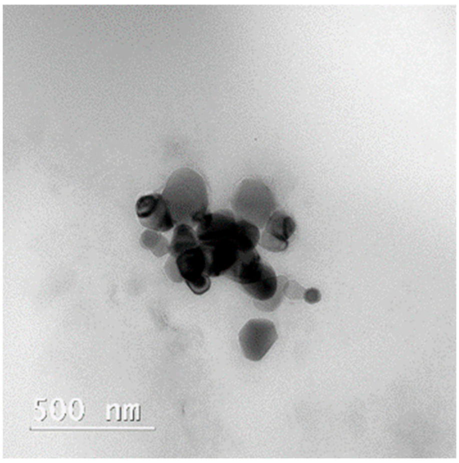

D

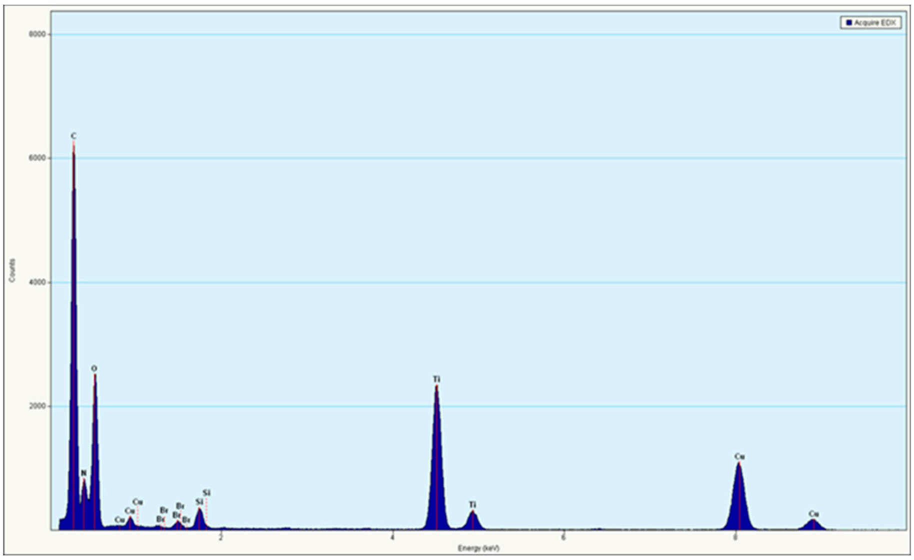

E

Figure S1 - TEM images of bentonite layers (A, C) and TiO_2 nanoparticles (A, B, D) and EDX spectra (E) of the hydrogel film IV.1

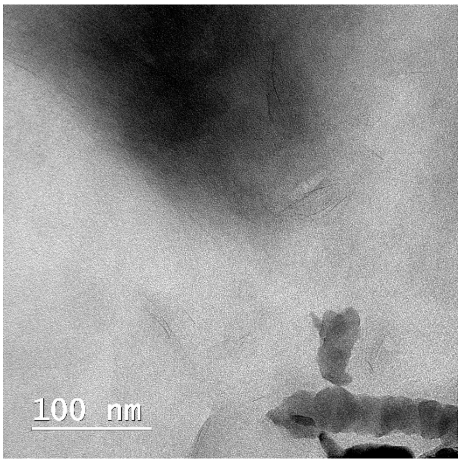

A

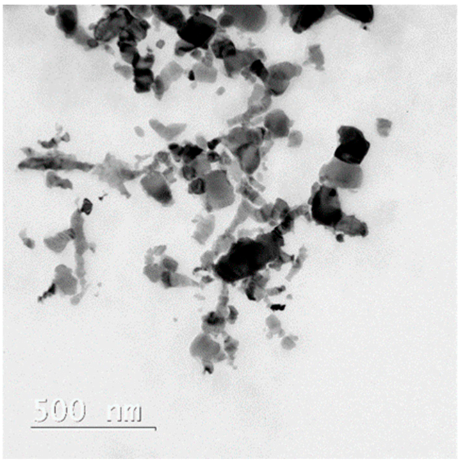

B

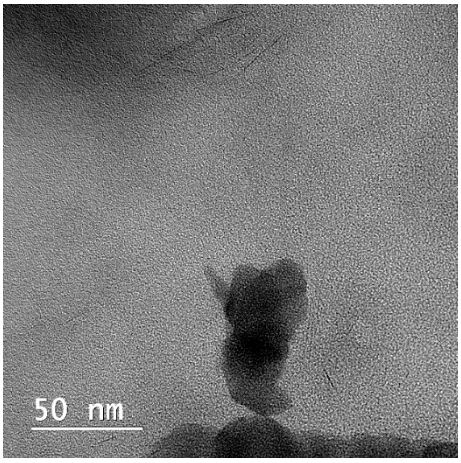

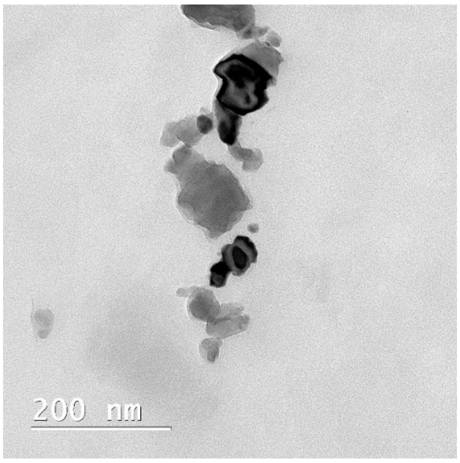

C

D

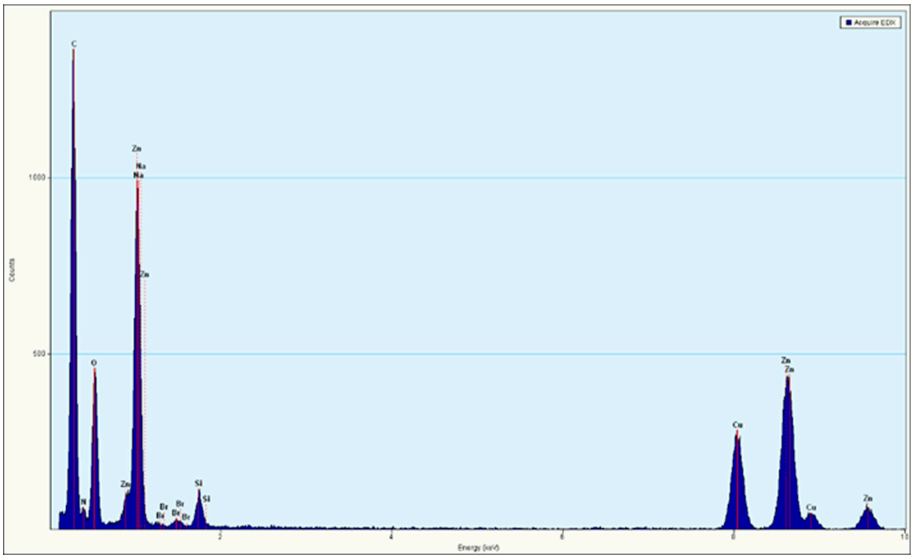

E

Figure S2 - TEM images of bentonite layers (A,C) and ZnO nanoparticles (A, B, C, D) and EDX spectra (E) of the hydrogel film IV.2

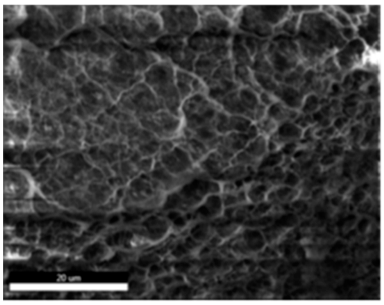

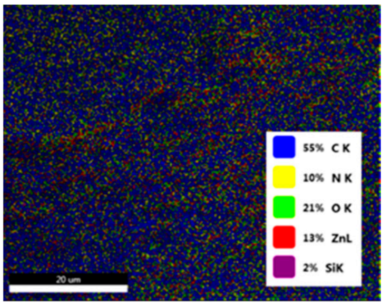

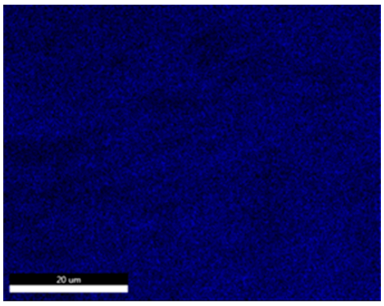

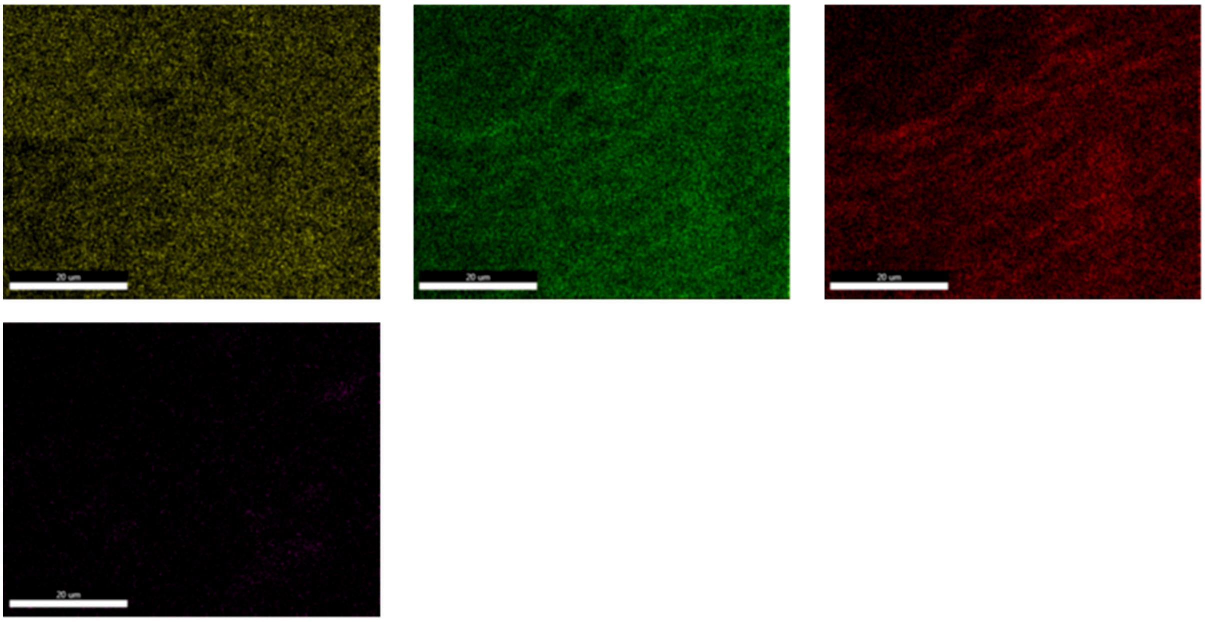

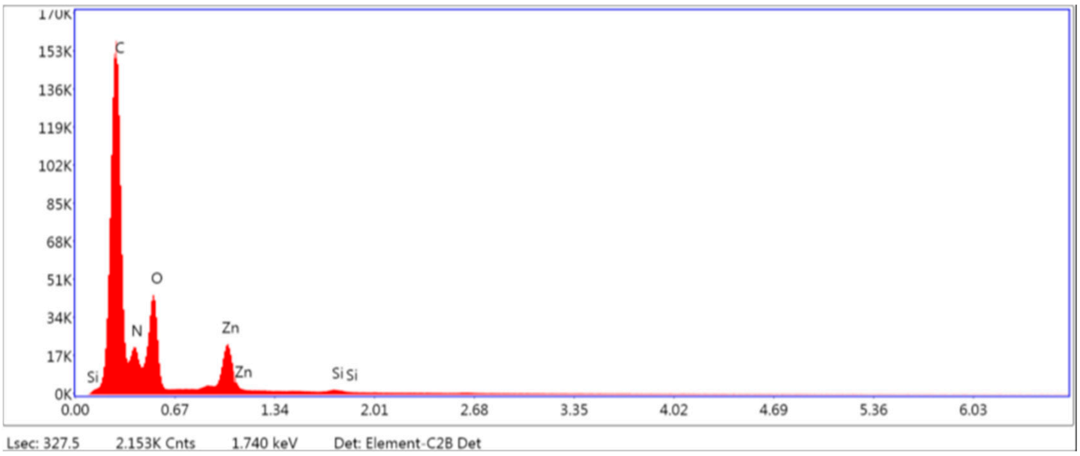

eZAF Smart Quant Results

Element	Weight %	Atomic %	Net Int.	Error %	Kratio	Z	R	A	F
C K	46.93	56.84	3604.60	5.49	0.3154	1.0555	0.9761	0.6367	1.0000
N K	19.42	20.18	479.20	9.60	0.0500	1.0240	0.9862	0.2513	1.0000
O K	22.30	20.28	940.40	8.68	0.0737	0.9970	0.9950	0.3315	1.0000
ZnL	10.75	2.39	373.70	2.78	0.0720	0.7135	1.1632	0.9390	0.9991
SiK	0.60	0.31	42.40	4.86	0.0049	0.8844	1.0329	0.9158	1.0043

Figure S3 – SEM-EDX mapping of sample IV

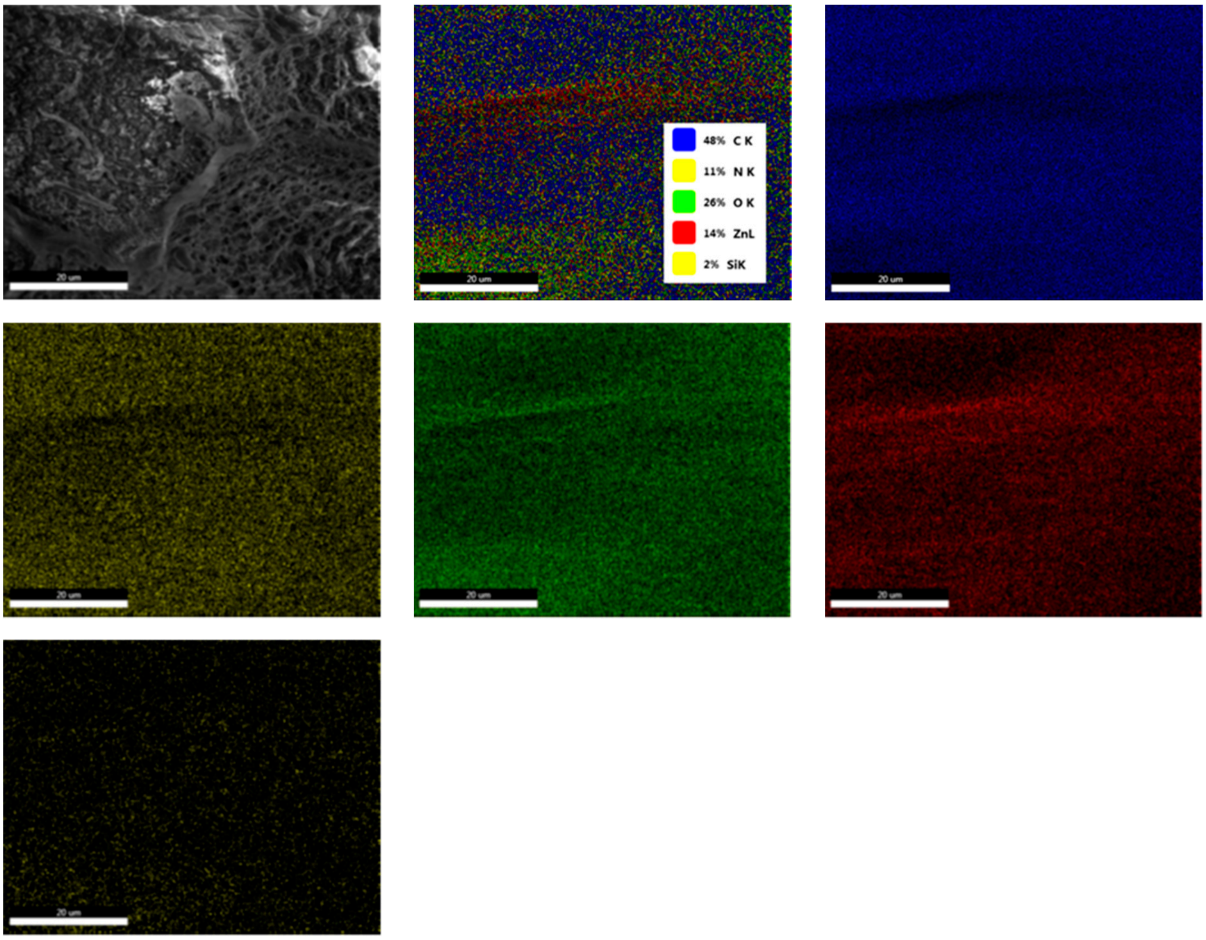

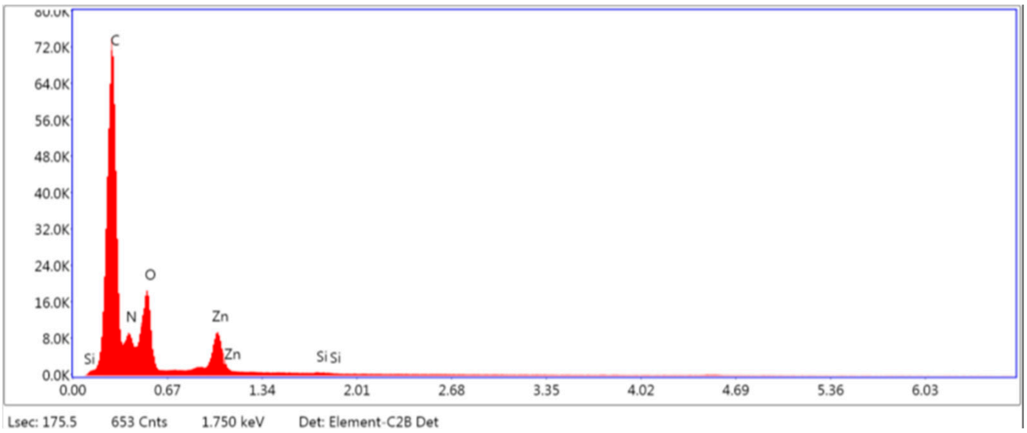

eZAF Smart Quant Results

Element	Weight %	Atomic %	Net Int.	Error %	Kratio	Z	R	A	F
C K	46.73	56.46	2988.60	5.39	0.3212	1.0545	0.9766	0.6517	1.0000
N K	19.93	20.65	411.80	9.64	0.0530	1.0231	0.9866	0.2600	1.0000
O K	22.46	20.37	781.70	8.75	0.0758	0.9961	0.9955	0.3387	1.0000
ZnL	10.52	2.33	296.80	2.92	0.0704	0.7128	1.1637	0.9405	0.9990
SiK	0.36	0.19	20.80	9.57	0.0030	0.8835	1.0332	0.9189	1.0043

Figure S4 – SEM-EDX mapping of sample IV.1

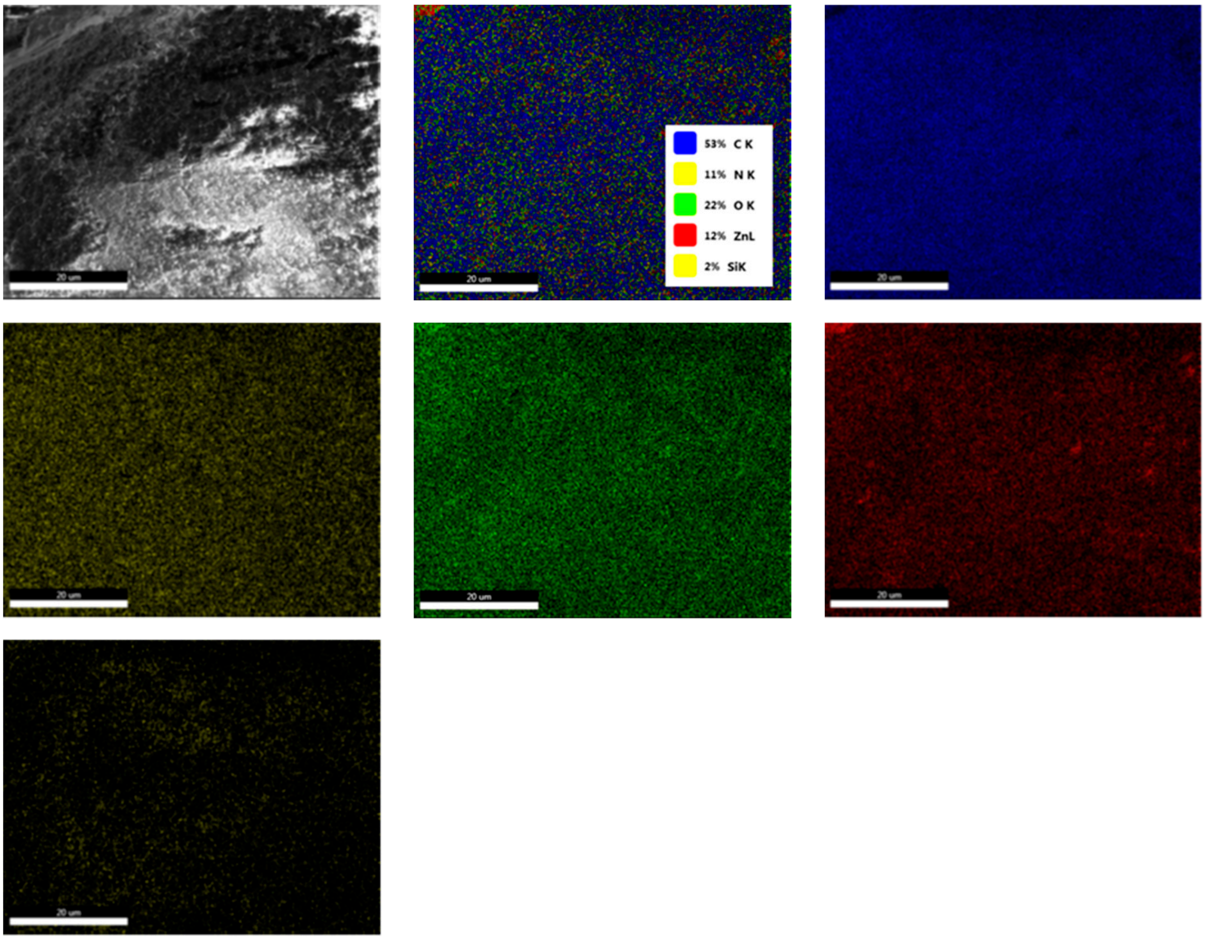

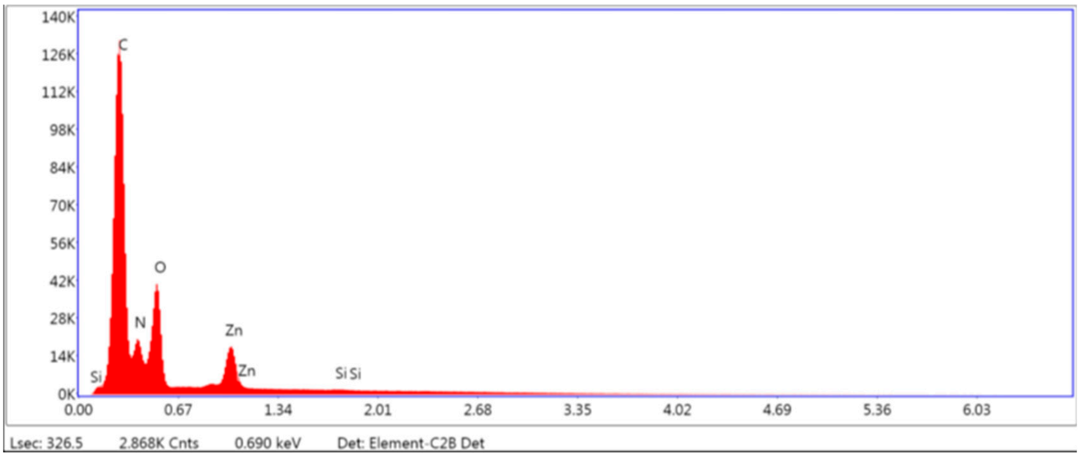

eZAF Smart Quant Results

Element	Weight %	Atomic %	Net Int.	Error %	Kratio	Z	R	A	F
C K	49.51	58.99	2790.10	5.14	0.3505	1.0500	0.9786	0.6742	1.0000
N K	17.46	17.84	292.60	9.84	0.0439	1.0186	0.9886	0.2471	1.0000
O K	23.59	21.10	704.30	8.68	0.0796	0.9917	0.9973	0.3401	1.0000
ZnL	9.43	2.06	226.90	3.15	0.0629	0.7095	1.1655	0.9401	0.9991
SiK	0.00	0.00	0.10	95.38	0.0000	0.8794	1.0346	0.9223	1.0045

Figure S5 – SEM-EDX mapping of sample IV.2

Table S2 – Concentrations of the representative elements in the nanocomposite hydrogels

Sample code	**Al** ppm, mg/kg	**Ti** ppm, mg/kg	**Zn** ppm, mg/kg
**IV**	559.60 ± 5.9%	12.44±2.9%	61449.57± 0.3%
**IV.1**	646.00 ± 2.9%	337.12± 3.9%	49524.46 ± 7.2%
**IV.2**	587.03 ± 10.9%	13.37± 11.8%	50787.42 ±14.0%

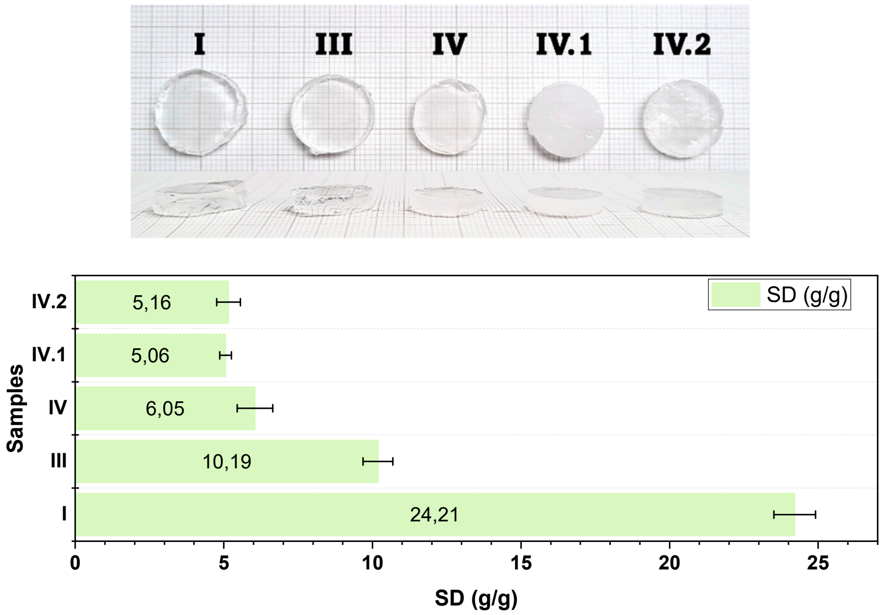

Figure S6 – Swelling degree at equilibrium

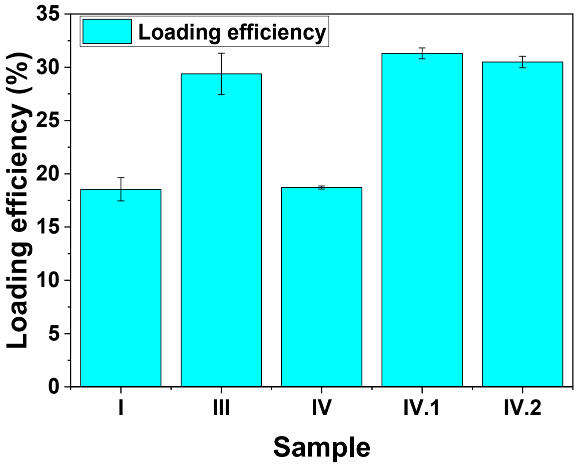

Figure S7 – Nafcillin loading efficiency at pH 7.4

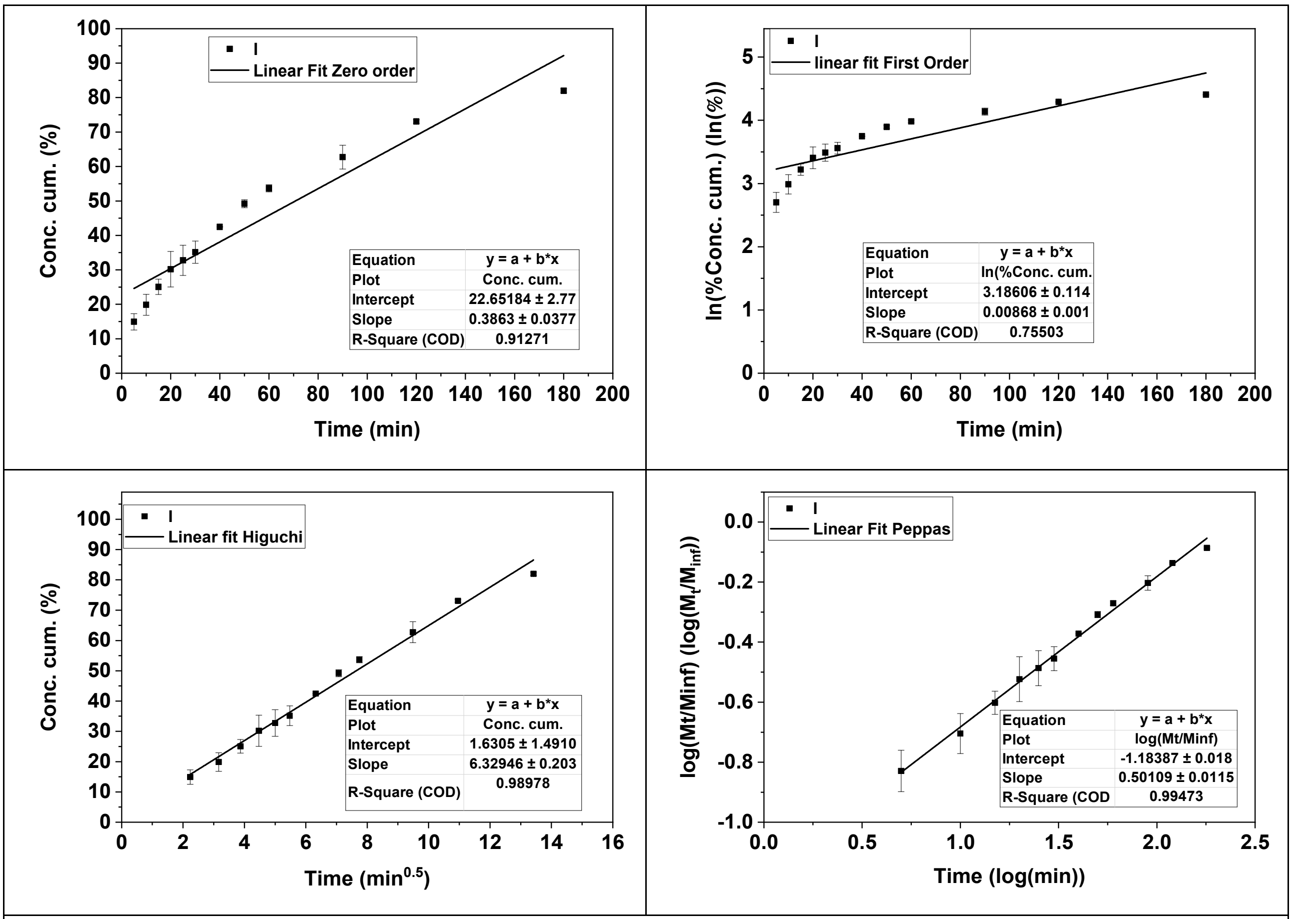
I
Linear Fit Zero order
Conc. cum. (%)
Time (min)
Equation | y = a + b*x
Plot | Conc. cum.
Intercept | 22.65184 ± 2.77
Slope | 0.3863 ± 0.0377
R-Square (COD) | 0.91271
I
linear fit First Order
ln(%Conc. cum.) (ln(%))
Time (min)
Equation | y = a + b*x
Plot | ln(%Conc. cum.
Intercept | 3.18606 ± 0.114
Slope | 0.00868 ± 0.001
R-Square (COD) | 0.75503
I
Linear fit Higuchi
Conc. cum. (%)
Time (min^0.5)
Equation | y = a + b*x
Plot | Conc. cum.
Intercept | 1.6305 ± 1.4910
Slope | 6.32946 ± 0.203
R-Square (COD) | 0.98978
I
Linear Fit Peppas
log(Mt/Minf) (log(Mt/Minf))
Time (log(min))
Equation | y = a + b*x
Plot | log(Mt/Minf)
Intercept | -1.18387 ± 0.018
Slope | 0.50109 ± 0.0115
R-Square (COD | 0.99473

I

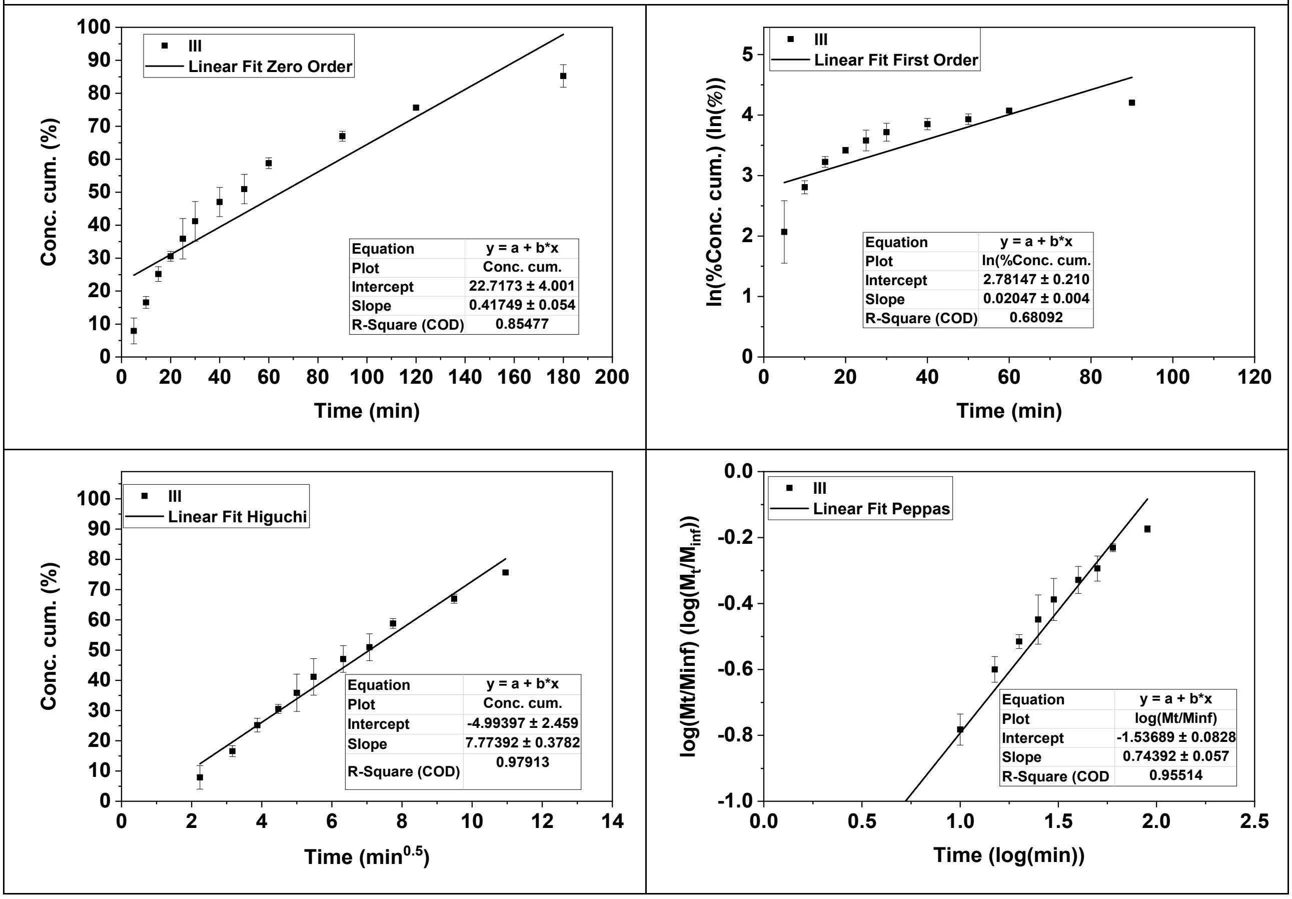
III
Linear Fit Zero Order
Conc. cum. (%)
Time (min)
Equation | y = a + b*x
Plot | Conc. cum.
Intercept | 22.7173 ± 4.001
Slope | 0.41749 ± 0.054
R-Square (COD) | 0.85477
III
Linear Fit First Order
ln(%Conc. cum.) (ln(%))
Time (min)
Equation | y = a + b*x
Plot | ln(%Conc. cum.
Intercept | 2.78147 ± 0.210
Slope | 0.02047 ± 0.004
R-Square (COD) | 0.68092
III
Linear Fit Higuchi
Conc. cum. (%)
Time (min^0.5)
Equation | y = a + b*x
Plot | Conc. cum.
Intercept | -4.99397 ± 2.459
Slope | 7.77392 ± 0.3782
R-Square (COD) | 0.97913
III
Linear Fit Peppas
log(Mt/Minf) (log(Mt/Minf))
Time (log(min))
Equation | y = a + b*x
Plot | log(Mt/Minf)
Intercept | -1.53689 ± 0.0828
Slope | 0.74392 ± 0.057
R-Square (COD | 0.95514

III

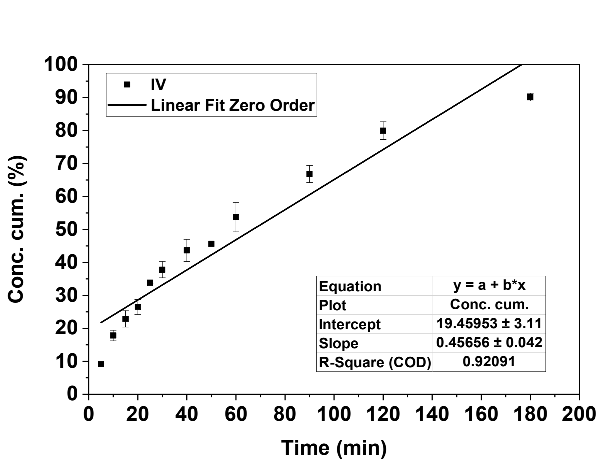
IV
Linear Fit Zero Order
Equation | y = a + b*x
Plot | Conc. cum.
Intercept | 19.45953 ± 3.11
Slope | 0.45656 ± 0.042
R-Square (COD) | 0.92091
Conc. cum. (%)
Time (min)

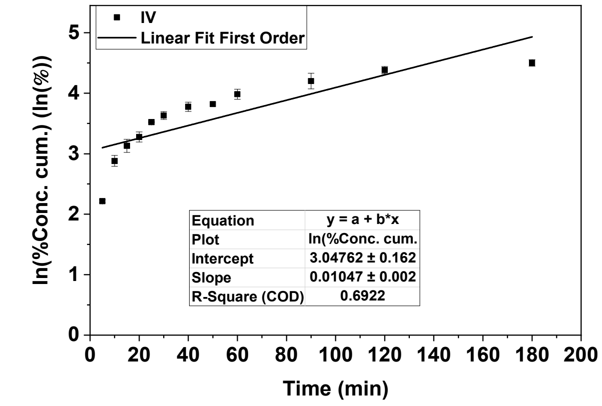
IV
Linear Fit First Order
Equation | y = a + b*x
Plot | ln(%Conc. cum.
Intercept | 3.04762 ± 0.162
Slope | 0.01047 ± 0.002
R-Square (COD) | 0.6922
ln(%Conc. cum.) (ln(%))
Time (min)

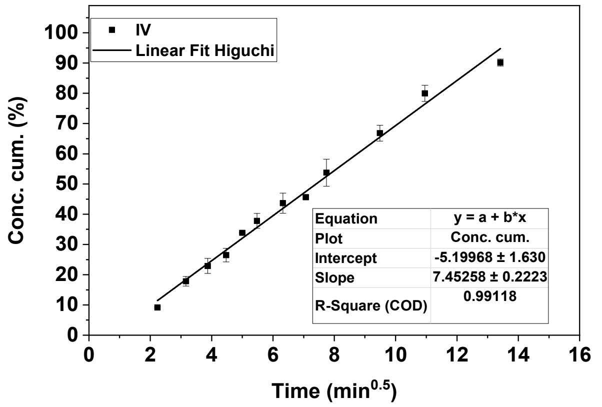
IV
Linear Fit Higuchi
Equation | y = a + b*x
Plot | Conc. cum.
Intercept | -5.19968 ± 1.630
Slope | 7.45258 ± 0.2223
R-Square (COD) | 0.99118
Conc. cum. (%)
Time (min^0.5)

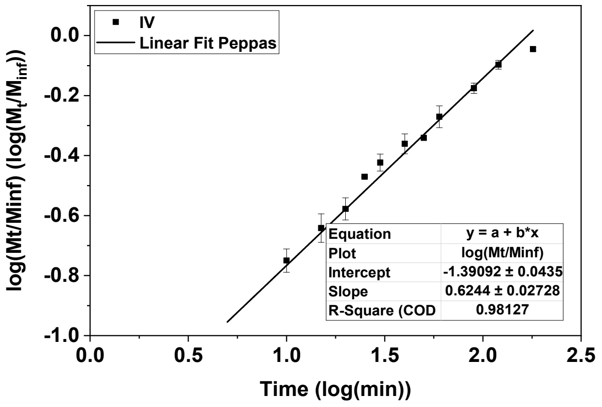
IV
Linear Fit Peppas
Equation | y = a + b*x
Plot | log(Mt/Minf)
Intercept | -1.39092 ± 0.0435
Slope | 0.6244 ± 0.02728
R-Square (COD | 0.98127
log(Mt/Minf) (log(Mt/Minf))
Time (log(min))

IV

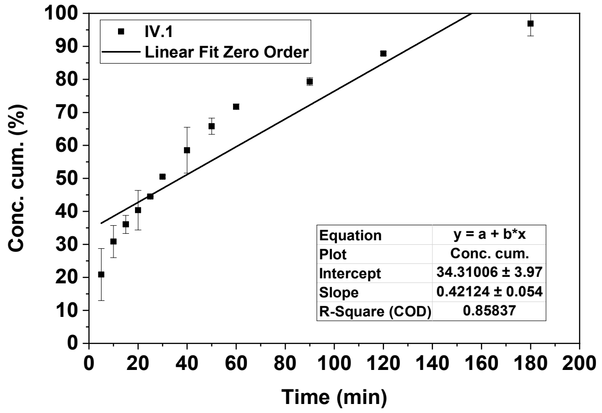
IV.1
Linear Fit Zero Order
Equation | y = a + b*x
Plot | Conc. cum.
Intercept | 34.31006 ± 3.97
Slope | 0.42124 ± 0.054
R-Square (COD) | 0.85837
Conc. cum. (%)
Time (min)

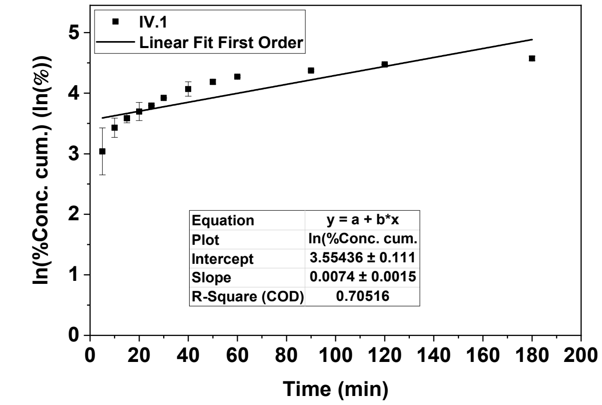
IV.1
Linear Fit First Order
Equation | y = a + b*x
Plot | ln(%Conc. cum.
Intercept | 3.55436 ± 0.111
Slope | 0.0074 ± 0.0015
R-Square (COD) | 0.70516
ln(%Conc. cum.) (ln(%))
Time (min)

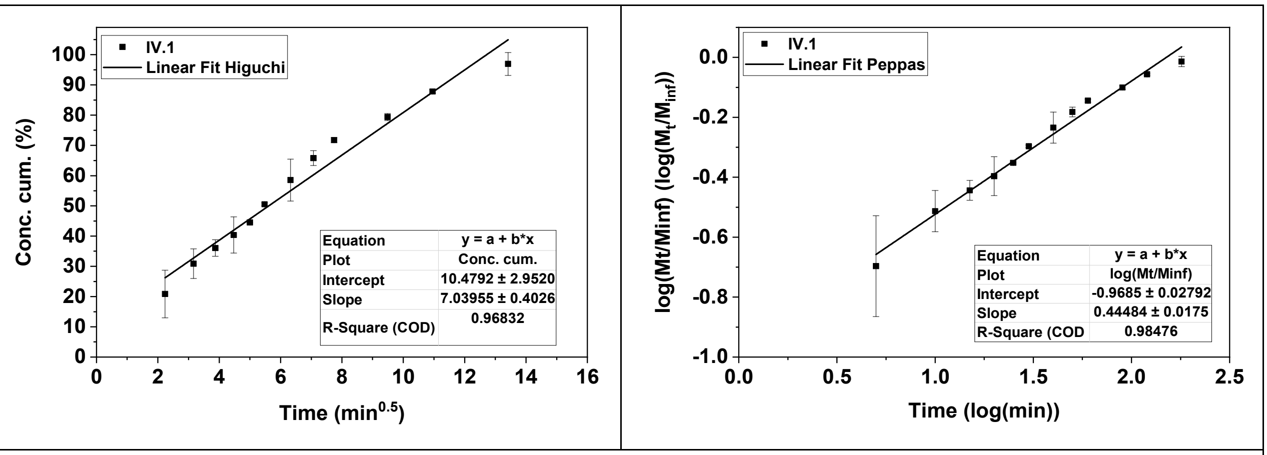

IV.1

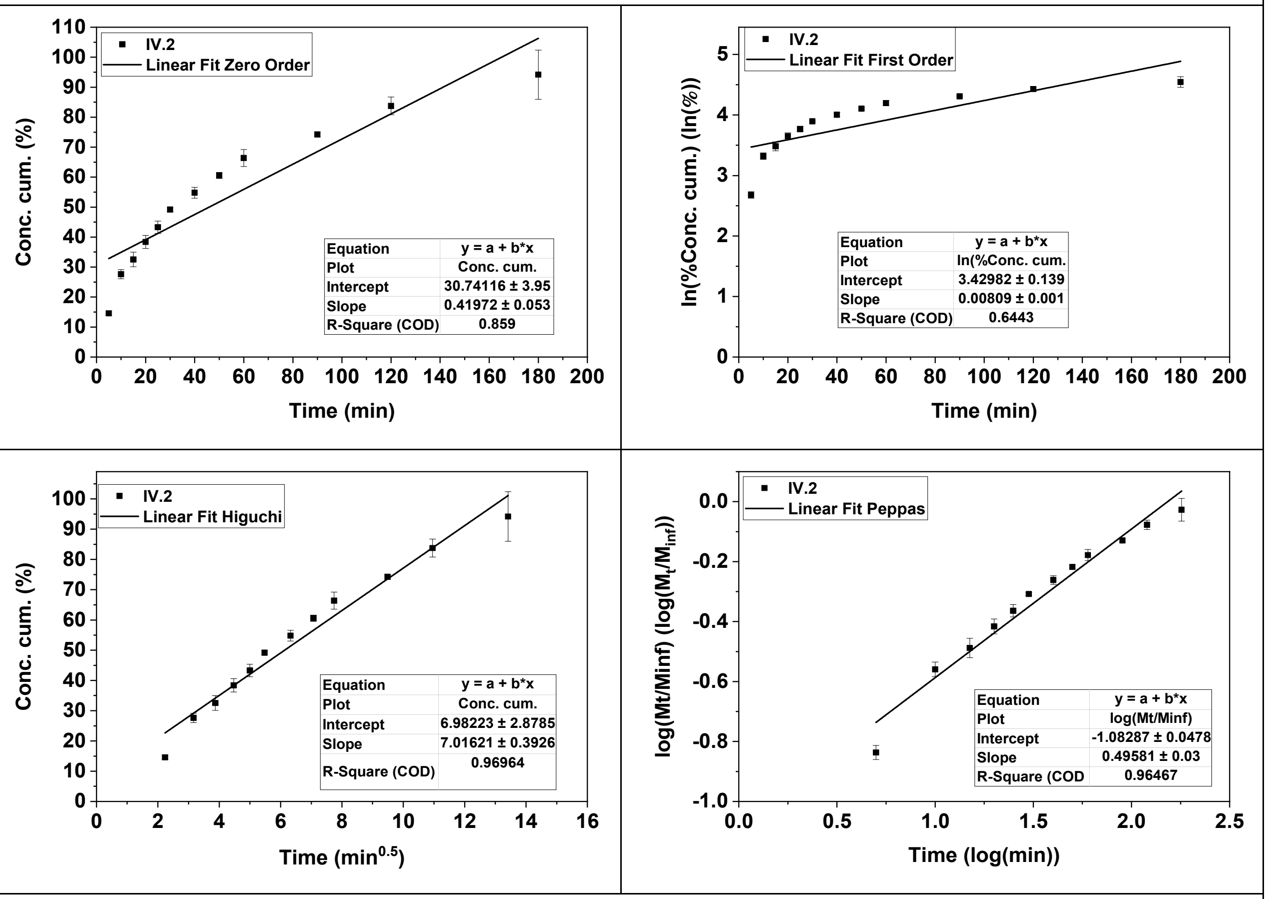

IV.2

Figure S8 – Nafcillin release - data fitting for samples I, III, IV, IV.1 and IV.2

Table S3 - Number of CFU when no drug was loaded in the nanocomposite hydrogel films

Samples	No. of CFU growth after 15 min		No. of CFU growth after 30 min		No. of CFU growth after 1 hour	
	S. aureus	*E. coli*	*S. aureus*	*E. coli*	*S. aureus*	*E. coli*
IV	2×10^7	5×10^7	3×10^7	12×10^7	4×10^7	9×10^7
IV.1	10^7	3×10^7	2×10^7	5×10^7	10^7	12×10^7
IV.2	3×10^7	9×10^7	4×10^7	10×10^7	**2×10^6**	**7×10^6**
PC *S.aureus*	5×10^7					
PC *E. coli*	10×10^7					
PC = control						

Table S4 - Minimal inhibitory concentration (MIC) and Minimal bactericidal concentration (MBC) values

Sample / Microorganism	*E. coli*		*S. aureus*	
	MIC (%)	MBC (%)	MIC (%)	MBC (%)
TiO_2 - NPs powder	> 2	--	> 2	--
ZnO - NPs powder	0.125	--	0.25	--

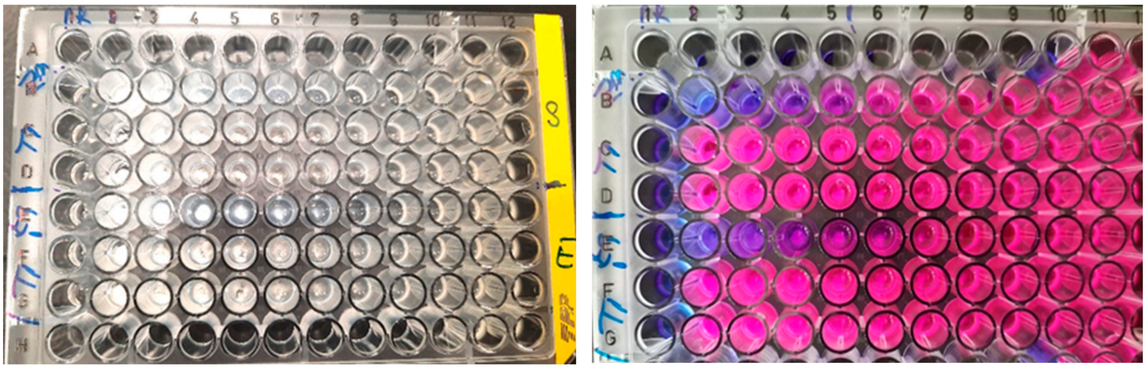

a b

Figure S9. - MIC determination observed from broth microdilution assay using MH broth and resazurin.

S. aureus ATCC 6538 (row B: ZnO; row C: TiO_2; row D: Positive Control S. aureus);
E. coli ATCC 8739 (row E: ZnO; row F: TiO_2 and row G: Positive Control E. coli);
Bk = first column (a)before resazurin (b)after resazurin
